# Discovery of Ketal‐Ester Ionizable Lipid Nanoparticle with Reduced Hepatotoxicity, Enhanced Spleen Tropism for mRNA Vaccine Delivery

**DOI:** 10.1002/advs.202404684

**Published:** 2024-10-10

**Authors:** Kai Lv, Zhenlei Yu, Jing Wang, Na Li, Apeng Wang, Tiezheng Xue, Qixin Wang, Yanqin Shi, Lu Han, Wei Qin, Jiaqi Gong, Huijuan Song, Tingting Zhang, Chunyan Chang, Hua Chen, Xijun Zhong, Jian Ding, Rui Chen, Mingliang Liu, Weiguo Zhang, Shan Cen, Yijie Dong

**Affiliations:** ^1^ Institute of Medicinal Biotechnology Chinese Academy of Medical Sciences and Peking Union Medical College Beijing 100050 China; ^2^ RinuaGene Biotechnology Co. Ltd Suzhou Jiangsu China

**Keywords:** biodegradability, ionizable lipid, mRNA delivery, organ tropism, toxicity

## Abstract

The safety and efficacy of the lipid nanoparticle (LNP) delivery system are crucial for the successful development of messenger RNA vaccines. We designed and synthesized a series of ketal ester lipids (KELs), featuring a biodegradable ketal moiety in the linker and ester segments in the tail. Through iterative optimization of the head and tail groups of KELs, we tuned the *p*Ka and molecular shapes, and identified (*4S*)‐KEL12 as a safe and efficient ionizable lipid for mRNA delivery. (*4S*)‐KEL12 LNP showed significantly higher delivery efficacy and lower toxicity than the DLin‐MC3‐DMA LNP. In comparison to SM‐102 LNP, (*4S*)‐KEL12 LNP exhibited better spleen tropism, reduced liver tropism, and hepatotoxicity. Additionally, (*4S*)‐KEL12 demonstrated good biodegradability following intramuscular or intravenous injection. Notably, (*4S*)‐KEL12 LNP encapsulated with a therapeutic mRNA cancer vaccine elicited robust cellular immune responses leading to substantial tumor regression along with prolonged survival in tumor‐bearing mice. Our results suggest that (*4S*)‐KEL12 LNP holds great promise for mRNA vaccine delivery. The comprehensive analysis of the structure‐activity relationship, toxicity, biodegradability, distribution, expression, efficacy, and stereochemistry of these LNPs will greatly contribute to the rational design and discovery of novel lipid‐based delivery systems.

## Graphical abstract

We developed (*4S*)‐KEL12, a biodegradable ketal ester lipid (KEL) with reduced liver tropism and lower toxicity, better spleen tropism, and excellent mRNA vaccine delivery efficacy, compared to MC3 and SM‐102 LNPs. (*4S*)‐KEL12 LNP induced strong immune responses, significant tumor regression, and prolonged survival in mice, showcasing its potential for mRNA vaccine delivery.

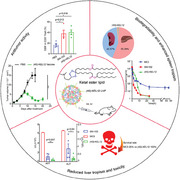

## 1 Introduction

During the coronavirus disease 2019 (COVID‐19) pandemic, the food and drug administration authorized emergency use for two messenger RNA (mRNA) vaccines, namely BNT162b2 and mRNA‐1273.^[^ ^1^ ^]^ These vaccines have demonstrated remarkable efficacy in reducing coronavirus transmission and preventing severe illness, thus playing a pivotal role in mitigating the impact of this challenging global health crisis. The rapid design and development of these two COVID‐19 mRNA vaccines signify a significant milestone in the field of RNA therapeutics.^[^ ^2^ ^]^ Moreover, mRNA‐based prophylactic and therapeutic vaccines have garnered substantial attention for their potential applications in numerous infectious diseases and cancers.^[^ ^3^ ^]^ For example, several lipid nanoparticle (LNP)‐based mRNA vaccines are currently undergoing clinical trials targeting influenza viruses, respiratory syncytial virus (RSV), cytomegalovirus, as well as cancers.^[^ ^2^ ^,^ ^4^ ^]^

Compared to traditional biopharmaceutical and vaccine technology, mRNA vaccines are reliant on the LNP delivery systems to overcome biological barriers and release the RNA payload into the cytosol of antigen presentation cells (APCs).^[^ ^5^ ^]^ LNPs are typically comprised of an ionizable amino lipid, a phospholipid, cholesterol (Chol), and a polyethylene glycol (PEG) lipid.^[^ ^6^ ^]^ The ionizable amino lipid plays a critical role in the drug delivery process. Such lipids contain pH‐sensitive amines that maintain a close to neutral surface charge at physiological pH, thereby reducing nonspecific lipid‐protein interactions and turning into cationic groups within the acidic endosomes after cellular uptake, thus facilitating endosomal destabilization and mRNA release into the cytosol.^[^ ^7^ ^]^

Safe and effective delivery systems are essential for the performance of LNP‐based RNA therapeutics. Currently, only three ionizable amino lipids are clinically approved for RNA therapies.^[^ ^7^, ^8^ ^]^ DLin‐MC3‐DMA is the first clinically approved RNA delivery lipid and has been used in siRNA therapy to knock down transthyretin in hepatocytes. However, DLin‐MC3‐DMA‐based‐LNP gave rise to mild to moderated local and systemic adverse events.^[^ ^7^, ^9^ ^]^ ALC‐0315 and SM‐102 are structurally similar lipids used in BioNTech and Moderna SARS‐CoV‐2 mRNA vaccines, respectively, proving to be generally safe in hundreds of millions of people.^[^ ^1^, ^10^ ^]^ However, data from human clinical trials and real‐world applications showed multiple side effects for both vaccines, some of which were suspected to be related to the delivery lipids.^[^ ^4c^ ^]^ Moreover, mice treated with si*Luc*‐ALC‐0315 had significantly higher levels of alanine transaminase (ALT) in serum, indicating potential hepatotoxicity.^[^ ^11^ ^]^ Further, SM‐102‐based mRNA‐LNP stimulated significant inflammation, contributing to some common side effects, at least partially attributable to the ionizable lipid itself.^[^ ^12^ ^]^ Since prophylactic vaccines, such as those against influenza or RSV, target a very large proportion of the human population, progress in improving the safety profile of the delivery system should benefit many individuals. Therefore, there remains an imminent necessity for novel ionizable lipids with enhanced safety and efficacy profiles for mRNA vaccine development.

Structurally, ionizable lipids typically consist of three components: (i) ionizable headgroups, (ii) linker groups, and (iii) hydrophobic tails.^[^ ^5^ ^]^ One strategy involves incorporating biodegradable moieties into the linker or tail of these lipids, which could enhance elimination kinetics and thereby mitigate potential adverse effects.^[^ ^13^ ^]^ It has been reported that lipids containing either a biodegradable primary ester in the tail or ketal ring in the linker region produced well‐tolerated LNP without significant alterations in ALT and aspartate transaminase (AST).^[^ ^14^ ^]^ This leads to a strategy of lipid design for improving safety profiles by incorporating different biodegradable moieties or their combination.

Furthermore, a successful mRNA vaccine requires effective LNP delivery into APCs such as dendritic cells (DCs).^[^ ^15^ ^]^ It has recently been demonstrated that SM‐102‐based EGFP mRNA‐LNPs led to efficient reporter expression in macrophages and DCs.^[^ ^16^ ^]^ However, most ionizable cationic lipid‐based LNPs including SM‐102 and ALC‐0315 have a strong hepatotropism influenced by dominant LNP interactions with liver‐targeting protein corona in the serum.^[^ ^17^ ^]^ Therefore, shifting the LNP tropism toward immune organs will have the potential to simultaneously enhance antigen presentation while reducing hepatotoxic LNP uptake in the liver. Spleen is an ideal site for initiating and amplifying antigen‐specific immune responses.^[^ ^18^ ^]^ Much effort has been made to achieve spleen‐selective or enrichment delivery,^[^ ^19^ ^]^ such as injection of negatively charged (SORT LNP) or large‐sized mRNA LNP.^[^ ^20^ ^]^ Nevertheless, caution must be taken when designing a lipid for better safety and efficacy due to the poorly understood structure‐activity relationship (SAR). Numerous studies showed that even a slight structural modulation of the ionizable lipid could result in dramatic changes in critical LNP physiochemical and biological attributes.^[^ ^17^, ^21^ ^]^ For example, incorporating biodegradable moieties may affect the *p*Ka value of ionizable lipids, which acts as a critical characteristic for optimizing LNP delivery efficacy.^[^ ^7^, ^22^ ^]^ Thus, the development of novel ionizable lipids must warrant both improved safety and efficacy profiles, which is the aim of this study.

We have designed a number of lipid series to obtain new cationic lipids with potentially improved safety features for mRNA delivery. Herein, we presented the design, synthesis, and biological profiles of a set of novel ketal ester lipids (KELs). These lipids were specifically engineered by integrating biodegradable ketal and ester groups into the structures of ionizable lipids, with the goal of enhancing their safety profiles. To achieve desirable delivery efficacy, we fine‐tuned the *p*Ka values and molecular shapes of the new KELs through iterative optimization of their head and tail groups. Among these lipids, (*4S*)‐KEL12 was identified as a safe and efficient ionizable lipid, supported by significantly reduced ALT and AST serum levels compared to SM‐102 after intravenous (IV) injection. (*4S*)‐KEL12 LNP afforded gene expression efficacy equivalent to SM‐102 in the spleen with significantly lower expression than SM‐102 in the liver. These results, together with consistent mRNA distribution data, suggested a favorable tropism shift toward immune organs. Additionally, (4S)‐KEL12 demonstrated good biodegradability following intramuscular (IM) or IV injection. Finally, we verified the therapeutic efficacy of an mRNA vaccine encoding human papillomavirus (HPV) antigens encapsulated by the (*4S*)‐KEL12 LNP in the TC‐1 tumor model. (*4S*)‐KEL12 vaccine resulted in robust cellular immune responses, significant tumor regression, and prolonged animal survival. In conclusion, our comprehensive analyses of the SAR, toxicity, biodegradability, efficacy, and stereochemistry of these lipids illustrate a strategy for rational design and discovery of novel lipid‐based delivery systems. The remarkable therapeutic efficacy, along with its favorable safety profile, suggests that (*4S*)‐KEL12 LNP holds great promise for future clinical applications.

## 2 Results and Discussion

### 2.1 Design of New Ketal Ester Lipids

**Figure 1:**
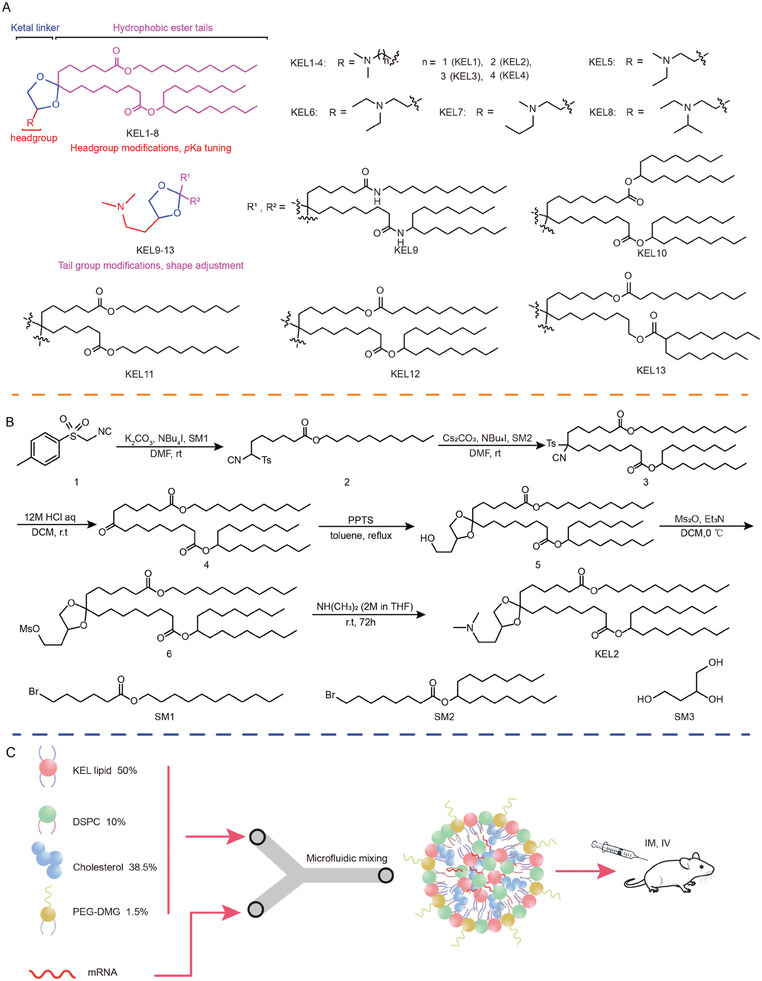
Structure, synthesis and in vivo screening of ketal ester lipids. A) Design of novel KEL lipids. B) Six‐steps linear synthesis of KELs. A representative synthesis scheme of KEL2 is shown. Changing the starting material SM1, SM2, SM3, and NH(NH_3_)_2_ with their analogues afforded KEL1‐13. C) Illustration of LNP formulation and screening method.

We hypothesized that lipids containing both the ester and ketal features could further improve their safety profiles. Accordingly, in this study, we developed a series of novel KELs (**Figure** **1**A). These newly designed KELs incorporated biodegradable ester groups and ketal groups in the tail and linker regions respectively, potentially offering improved safety profiles.

The *p*Ka value of ionizable lipids is a critical characteristic for optimizing LNP delivery efficiency.^[^ ^7^, ^22^ ^]^ An apparent *p*Ka within the range of 6–7 represents an optimal window for highly efficient LNP delivery.^[^ ^21^, ^23^ ^]^ Each lipid possesses a distinct *p*Ka value that can be modified by altering its headgroup, linker structure, and hydrophobic tail composition accordingly. In addition, following the molecular shape hypothesis outlined several decades ago, increased branching has been postulated to facilitate endosomal escape.^[^ ^7^, ^24^ ^]^ Therefore, we performed iterative optimization on both the head group (KEL1‐8) and tail group (KEL9‐13) to fine‐tune their *p*Ka values and molecular shapes with the aim of obtaining lipids that exhibit enhanced delivery efficiency.

### 2.2 Synthesis of the Ketal Ester Lipids

The synthesis of ketal ester lipids was accomplished through a six‐step process with purity exceeding 95%. The typical synthetic route for KEL2 is illustrated in Figure 1B. Sequential nucleophilic substitution reactions involving tosymethyl isocyanide with bromide SM1 and SM2, in the presence of K_2_CO_3_ and Cs_2_CO_3_, respectively, resulted in compound **3**. Subsequent hydrolysis of compound **3** under acidic conditions yielded ketone **4**. The condensation reaction between ketone 4 and butane‐1,2,4‐triol SM3 was catalyzed by pyridinium *p‐*toluenesulfonate (PPTS), employing a Dean‐Stark tube for efficient water removal, leading to the pivotal intermediate **5**. Mesylation of compound **5** followed by nucleophilic substitution resulted in KEL2. By modifying the raw materials (SM1‐3 and dimethylamine) in this synthetic pathway, it is feasible to synthesize all ketal ester lipids.

### 2.3 Screen and Structure‐Activity Relationship of Novel Ketal Lipids

Following the formulation methods of SpikeVax and Onpattro, all KEL LNPs were formulated with standard helper lipids: DSPC, Chol, and DMG‐PEG2000 at a molar ratio of Ionizable lipid/DSPC/Chol/DMG‐PEG2000 50:10:38.5:1.5, and at an ionizable lipid to RNA charge ratio (N/P) of 6.^[^ ^25^ ^]^ The physicochemical properties of the newly formulated LNPs were characterized and presented in **Table** **1**. Particle size and polydispersity index (PDI) were determined by dynamic light scattering (DLS), apparent *p*Ka was assessed using 6‐(*p‐*toluidino)‐2‐naphthalenesulfonic acid (TNS) dye‐binding assay, while encapsulation efficiency was evaluated by a RiboGreen accessibility assay. Overall, all LNPs exhibited high encapsulation efficiency (>85%), with most having hydrodynamic diameters below 100 nm and a narrow size distribution with PDI < 0.2, similar to DLin‐MC3‐DMA.

**Table 1:** Physiochemical properties of Ketal Ester Lipids LNPs.

Compd.	Encapsulation efficiency (%)	Size (nm)	PDI	*p*Ka
DLin‐MC3‐DMA	96.7	72.70	0.15	6.33
KEL1	95.0	76.72	0.12	5.72
KEL2	92.3	70.27	0.03	6.88
KEL3	89.3	98.15	0.16	6.86
KEL4	91.0	118.1	0.16	7.40
KEL5	96.5	78.44	0.07	6.86
KEL6	97.5	80.34	0.06	6.89
KEL7	97.2	61.73	0.10	5.91
KEL8	97.6	71.68	0.03	6.17
KEL9	98.2	125.0	0.05	7.03
KEL10	96.5	69.23	0.10	6.09
KEL11	93.3	110.3	0.34	6.88
KEL12	94.9	89.54	0.04	6.93
KEL13	91.0	99.68	0.09	6.44

**Figure 2:**
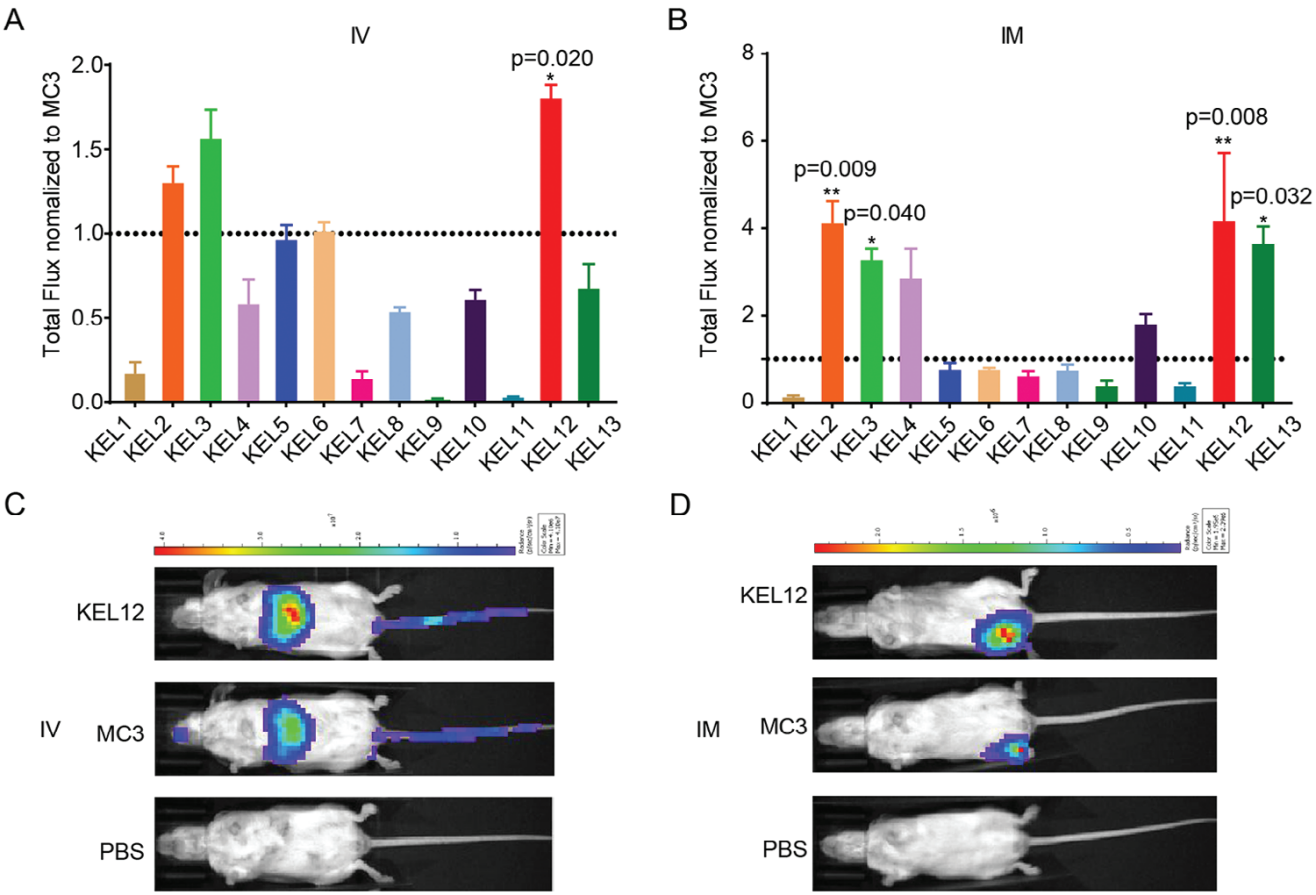
Screening of KEL LNPs. A‐B) Bar graphs of total bioluminescent flux in the whole body of female Balb/c mice for different KEL LNPs, normalized by DLin‐MC3‐DMA (MC3) LNP expression (n = 3–5/group). A) IV, 1 mg k^−1^g; B) IM, 0.25 mg k^−1^g. C‐D) Representative whole‐body bioluminescence images of luciferase signal in mice after injected with *m*Luci‐KEL12 LNP or DLin‐MC3‐DMA LNP, or PBS only as the negative control. Data were presented as mean ± SEM. (A and B) Statistical significance was calculated by using One‐Way ANOVA test followed by the Dunnett's post‐hoc test. * *p* < 0.05, ** *p* < 0.01.

Subsequently, the formulated KEL LNPs were evaluated for their in vivo delivery efficiency (Figure 1C). Wild‐type female Balb/c mice were administered with a single dose of LNPs containing firefly luciferase mRNA (*m*Luci) via IV or intramuscular (IM) injection (**Figure** **2**A,B), followed by whole‐body luciferase activity assay. A control group receiving DLin‐MC3‐DMA‐based LNP was included in each experiment to facilitate comparisons across experiments.

Initially, we studied the impact of headgroups on the delivery efficiency. As shown in Figure 1A, KEL1‐4 with identical hydrophobic tails and dimethyl groups were first synthesized. KEL2 and KEL3 LNPs with two or three carbons exhibited higher potency than KEL1 and KEL4 with one or four carbons. Importantly, following IM administration, both KEL2 and KEL3 LNPs demonstrated significantly higher levels of luciferase activity than the DLin‐MC3‐DMA LNP (Figure 2A,B). It should be noted that the *p*Ka values of both KEL1 and KEL4 are outside the reported optimal range (*p*Ka: 6–7), which may contribute to their lower potency. We then explored KEL5‐8 with sterically large moieties at the head group of KEL2. However, KEL5‐8 LNPs showed reduced potency compared to KEL2 LNP following both IV and IM administration, suggesting that the dimethyl amine group is optimal for the head group.

Next, we investigated KEL9‐13 with distinct hydrophobic tails to explore their impacts on efficiency (Figure 1A). Notably, the tail moiety influences not only the effectiveness of delivery but also the LNP distribution pattern. For instance, Xu and colleagues reported lung‐selective mRNA delivery using so‐called “N”‐series LNPs.^[^ ^17b^ ^]^ Inspired by this strategy, we synthesized KEL9 with an amide bond. However, KEL9 exhibited significantly reduced delivery potency, potentially due to its higher *p*Ka value (*p*Ka = 7.03). Drawing inspiration from the tail feature of ALC‐0315,^[^ ^26^ ^]^ we further designed KEL10 with double‐branched tails. Unfortunately, its potency diminished compared to KEL2. Additionally, our investigation of the double primary ester lipid‐based LNP (KEL11) revealed a further decrease in potency compared to KEL10 (Figure 2A,B). It is worth noting that both KEL9 and KEL11 LNPs displayed larger particle sizes (>100 nm), which might be associated with their reduced delivery efficiency. By altering the ester direction of KEL2, two new lipids, namely KEL12 and KEL13, were obtained. The IM administration of these novel LNPs (KEL12 and KEL13) demonstrated significantly higher luciferase activity than DLin‐MC3‐DMA LNP. Interestingly, KEL12 but not KEL13 LNP also exhibited significantly higher luciferase activity than DLin‐MC3‐DMA LNP by IV (Figure 2A,C). Together, these results indicated that only some specific modulations of the tail structure support efficient delivery.

We observed a correlation between KEL pKa and LNP potency, consistent with previous studies in the field (Figure 2 and Table 1).^[^ ^22^, ^23^ ^]^ Generally, LNPs with *p*Ka values outside the range of 6–7, such as KEL1 (*p*Ka 5.72), KEL7 (*p*Ka 5.91), and KEL9 (*p*Ka 7.03), demonstrated lower delivery efficiency for both IV and IM administration. LNPs with *p*Ka between 6.0‐6.5 (KEL8, KEL10, and KEL13) showed lower potency than those with *p*Ka of 6.7‐7.0 (KEL2, KEL3, KEL5, KEL6, and KEL12) after IV administration. Interestingly, as an exception, KEL4 exhibited considerable potency with a high pKa at 7.4. In terms of LNP shapes, LNPs featuring one linear and one branched tail (KEL2, KEL12, and KEL13) exhibited higher potency than LNPs with either double branched tails (KEL10) or double linear tails (KEL11) after IM or IV administration. The apparently small difference in ester direction between KEL12 and KEL13 resulted in some notable changes in not only LNP potency (Figure 2), but also several physiochemical features of LNP, including PDI, encapsulation efficiency, particle size, and pKa (Table 1). Specifically, KEL12 is better in PDI and encapsulation efficiency than KEL13. Together, we decided to prioritize further investigations on KEL12 LNP in this study thanks to its best overall performance in delivery potency and physiochemical characteristics.

### 2.4 The Impact of Stereochemistry on the Delivery Efficacy of KEL12

**Figure 3:**
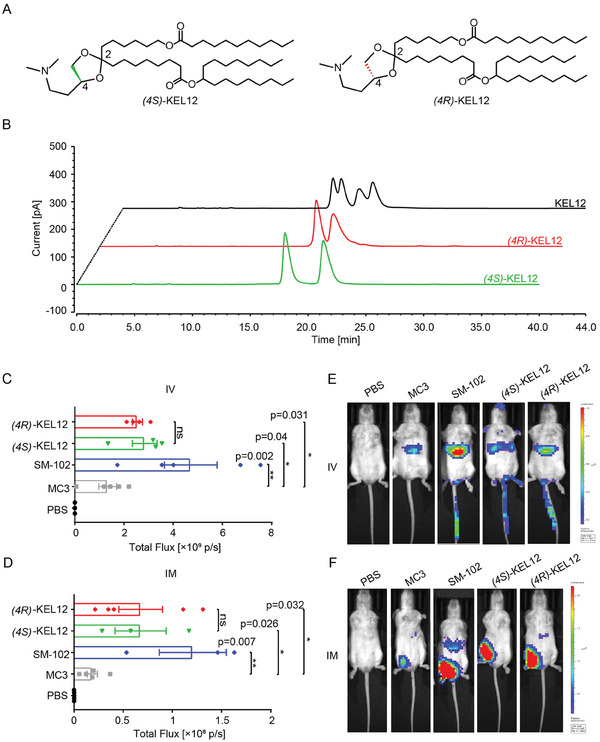
The impact of stereochemistry on the delivery efficiency. A) Structures of (*4S*)‐KEL12 and (*4R*)‐KEL12. B) HPLC spectra of (black) KEL12, (red) (*4R*)‐KEL12, and (green) (*4S*)‐KEL12. C‐D) Bar graphs of total bioluminescent flux for different LNPs in the whole body of female Balb/c mice. MC3 represents DLin‐MC3‐DMA. C) IV, 1 mg k^−1^g; D) IM, 0.25 mg k^−1^g. Data were presented as mean ± SEM (n = 4). Statistical significance was calculated by using One‐Way ANOVA test followed by the Dunnett's post‐hoc test, * *p* < 0.05, ** *p* < 0.01. E‐F) Representative whole‐body bioluminescence images of luciferase signal in mice for different LNPs by (E) IV or (F) IM administration (n = 3–5/group). PBS only served as the negative control.

Stereochemistry can generally influence the pharmacokinetics, safety, and efficacy of small‐molecule compounds.^[^ ^27^ ^]^ Further, stereochemistry‐dependent interactions between LNP and target cells can affect mRNA delivery efficiency.^[^ ^28^ ^]^ Since lipid KEL12 contains two chiral carbon atoms, we investigated the impact of chirality on delivery efficiency by obtaining its isomers. We synthesized (*4S*)‐KEL12 and (*4R*)‐KEL12 using a synthetic route outlined in Figure 1B with (*S*)‐SM3 and (*R*)‐SM3 as starting materials, respectively (**Figure** **3**A,B), since direct HPLC chiral separation failed to yield all four isomers of KEL12 (Figure 3B). Comparative analysis revealed improved delivery efficacy for all isomeric LNPs compared to DLin‐MC3‐DMA LNP for both IV and IM (Figure 3C‐F). However, no significant difference was observed between (*4S*)‐KEL12 and (*4R*)‐KEL12. Additionally, HPLC chiral separation of (*4S*)‐KEL12 yielded another two isomers, (*4S, 2R*)‐KEL12 and (*4S, 2S*)‐KEL12 specifically, without significant difference in delivery potency (Figure S1A,B).

*(4S*)‐KEL12 was chosen for further investigation due to easy access to raw materials. To determine its delivery efficacy, (*4S*)‐KEL12 LNP encapsulating a different mRNA reporter, *hEPO*, was assessed for its ability to express the hEPO proteins at various time points following IV or IM administration, respectively. As depicted in Figure S2, consistent with the findings from the *m*Luci investigation, (*4S*)‐KEL12 LNP exhibited a plasma hEPO protein level that is higher than DLin‐MC3‐DMA LNP but lower than SM‐102 LNP.

### 2.5 Expression Tropisim of (4S)‐KEL12 LNP

**Figure 4:**
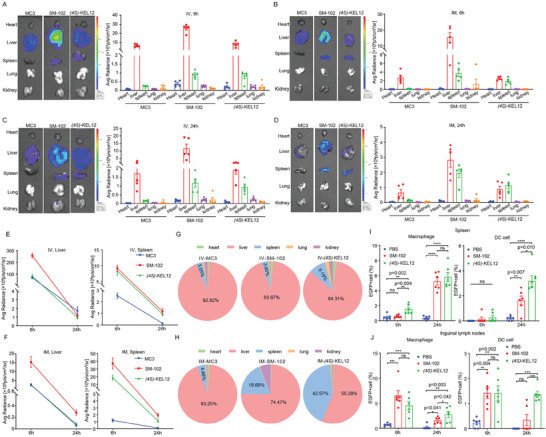
Organ and tissue tropism of (*4S*)‐KEL12 LNP. A‐D) Bioluminescent flux (left) images and (right) quantifications in various isolated major organs of female Balb/C mice after IV (1 mg/Kg) or IM (0.25 mg/Kg) injection of *mLuci* mRNA encapsulated by different LNPs at 6 h (A‐B) and 24 h (C‐D). MC3, SM‐102, and(*4S*)‐KEL12 LNPs were compared. E‐F) Bioluminescent flux in liver and spleen after mRNA‐LNP administration by (E) IV or (F) IM. MC3, SM‐102, and (*4S*)‐KEL12 LNPs were compared. G‐H) Percentage of fluorescence intensity in major organs after *mLuci* LNP injections by (G) IV or (H) IM. I‐J) Expression of *EGFP* mRNA KEL LNP in DC cells (CD11c^+^, CD45^+^) and macrophages (CD11b^+^, CD45^+^) from (I) spleen and (J) inguinal lymph nodes as measured by FACS after IV injection. MC3 represents DLin‐MC3‐DMA. Data were plotted as mean ± SEM (n = 5–10). Statistical significance was calculated by using One‐Way ANOVA test followed by the Student‐Newman‐Keuls post‐hoc test, ns *p* > 0.05, * *p *< 0.05, ** *p* < 0.01, *** *p* < 0.001 (I and J).

To further understand the profile of the new KEL LNP, we investigated the organ expression tropism of (*4S*)‐KEL12 LNP while using both DLin‐MC3‐DMA LNP and SM‐102 LNP as controls. After IM injection of 0.25 mg/Kg or IV injection of 1.0 mg/Kg *m*Luci LNPs, mouse organs were harvested after 6 h and 24 h, respectively. The tissues were subsequently measured for the average luminescence for each organ. As demonstrated by representative images and data quantifications, for all the LNPs, luciferase activity was predominantly observed in the liver and significantly in the spleen, with minimal translation in other organs, regardless of administration routes (**Figure** **4**A‐D).

In the liver, (*4S*)‐KEL12 LNP exhibited comparable expression to DLin‐MC3‐DMA LNP, yet significantly lower than SM‐102 LNP (left panels, Figure 4E,F). To better understand the reduced liver tropism for KEL12 LNP compared to SM‐102 LNP, we delivered KEL12 or SM‐102 LNPs encapsulating the CRE recombinase mRNA by IM in a *Rosa26* transgenic mice overexpressing a *CAG‐LSL‐tdTomato‐WPRE‐polyA* transgene (Figure S3). The CRE recombinase expression can remove a stop codon to activate tdTomato. FACS analysis on single cell suspension showed that SM‐102 LNP led to significantly higher frequencies of tdTomato^+^ Kupffer cells and tdTomato^+^ endothelial cells than KEL12 LNP, indicating limited liver tropism at the cell type level for the KEL12 LNP after IM delivery.

In addition, protein corona can influence the distribution and expression of LNPs. Thus, we incubated *mLuci* LNPs with mouse plasma before evaluating their expression in the Hepa1‐6 liver cancer cell line (Figure S4). LNPs incubated with plasma showed higher expression than untreated LNPs, indicating that factors in the mouse plasma enhanced luciferase expression in Hepa1‐6 cells. Notably, regardless of plasma incubation, SM‐102 LNP demonstrated higher levels of expression than the KEL12 LNP. This result suggested an overall better ability of expression of SM‐102 LNP than of KEL12 LNP in the liver cancer cell line under the influence of protein corona in mouse plasma. Moreover, we identified 327 and 326 proteins bound on the SM102 LNP and (*4S*)‐KEL12 LNP through proteomics analysis, respectively (Dataset S1). Among these, 224 proteins were common to both LNPs, while each LNP had around 100 unique proteins. These unique proteins may contribute to the different expression tropism of the two LNPs. Quantitative analysis and further characterization of these unique proteins may elucidate the mechanisms behind the expression tropisms in future studies.

In the spleen, (*4S*)‐KEL12 LNP showed higher expression than DLin‐MC3‐DMA LNP, and is comparable to SM‐102 LNP for IV but somewhat lower than SM‐102 LNP for IM (right panels, Figure 4E,F). To further compare the specific immune cell types transfected by (*4S*)‐KEL12 LNP or control SM‐102 LNP, LNPs encapsulated with *EGFP*‐mRNA were administered via IV injection, followed by flow cytometry analysis of single‐cell suspensions from harvested spleens and inguinal lymph nodes. In the spleen, (*4S*)‐KEL12 LNP exhibited significantly higher efficiency than SM‐102 LNP in DCs in the spleen at 24 hrs (right, Figure 4I). Moreover, both (*4S*)‐KEL12 and SM‐102 LNPs showed significantly higher ratios of EGFP‐positive macrophages and DCs than the PBS control at 24 h, as well as increases in the ratios of EGFP‐positive macrophages and DCs from 6 hr to 24 hr. In the inguinal lymph nodes, for macrophages, both LNPs displayed peak transfection efficiencies at 6 hr, followed by obvious decreases at 24 hr (left, Figure 4J). For DCs in inguinal lymph nodes, however, the two LNPs demonstrated differential expression patterns. Specifically, the SM‐102 LNP group showed a reduction from 6 hr to 24 hr, but the (*4S*)‐KEL12 LNP group maintained the ratio roughly unchanged over time (right, Figure 4J). Given that DCs are professional APCs that can activate antigen‐specific T cells and induce effective antitumor immune responses,^[^ ^15^ ^]^ (*4S*)‐KEL12 LNP showed advantageous features for DC expression over SM‐102 LNP. These results provided a high‐resolution picture of the improved immune organ tropism of (4S)‐KEL12 LNP. Overall, (*4S*)‐KEL12 LNP demonstrated enhanced expression tropism in the spleen relative to the liver compared to DLin‐MC3‐DMA and SM‐102 LNPs (Figure 4G,H), indicating the potential of (*4S*)‐KEL12 LNP for extrahepatic delivery, specifically for spleen‐related therapies.

### 2.6 Toxicity of (4S)‐KEL12 LNP

**Figure 5:**
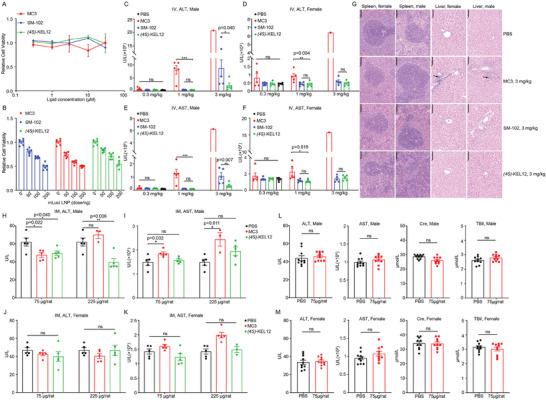
Toxicity of (*4S*)‐KEL12 LNP. A) The cell viability of 293T cells treated with lipid compounds. B) The cell viability of 293T cells treated with increasing amounts of formulated *m*Luci LNPs. C‐F) Serum levels of liver enzymes in Sprague‐Dawley rats following IV injection for 24 hours. ALT, alanine aminotransferase; AST, aspartate aminotransferase. C‐D) male rats. E‐F) female rats. G) Histological assessments of liver and spleen after IV injection of 3 mg k^−1^g *m*Luci LNP. Scale bar = 100 µm. H‐K) Serum levels of liver enzymes in Sprague‐Dawley rats 24 hours after a single IM injection. L‐M) Serum levels of liver enzymes in (L) male and (M) female Sprague‐Dawley rats three days after receiving IM injections once a week for four weeks. ALT, AST, Creatinine (Cre), and total bilirubin (TBil). MC3 represents DLin‐MC3‐DMA. For statistical analysis, data were presented as mean ± SEM (n = 5–10). Statistical significance was calculated by using One‐Way ANOVA test followed by the Student‐Newman‐Keuls post‐hoc test (I and J). ns, *p* > 0.05; **, *p* < 0.01; ***, *p* < 0.001.

One primary goal of developing ketal ester lipids was to enhance their biocompatibility. Therefore, we first assessed the cytotoxicity of (*4S*)‐KEL12 lipid alone and its *m*Luci‐LNP in cultured 293T cells, controlled by DLin‐MC3‐DMA and SM‐102. None of the lipids exhibited significant toxicity in 293T cells by themselves, with over 80% cell viability at 50 µM concentration in CCK8 assays (**Figure** **5**A). In addition, *m*Luci‐LNPs displayed some dose‐dependent cytotoxicity, with 50% cell viability observed at 200 ng mRNA per well in our experimental setting (Figure 5B). However, no significant differences were observed among (*4S*)‐KEL12, DLin‐MC3‐DMA, and SM‐102 or their respective *m*Luci‐LNPs in the in vitro cytotoxicity assay (Figure 5A,B).

Moreover, a dose escalation toxicity study was conducted in male and female Sprague‐Dawley rats (n = 5/group). The *m*Luci‐LNP formulations were administered via a single IV injection at dose levels of 0.3, 1.0, and 3.0 mg k^−1^g (Figure 5C‐F). At a dose of 1.0 mg k^−1^g, DLin‐MC3‐DMA LNP induced a significant increase in the ALT and AST levels compared to PBS‐treated male or female rats, suggesting hepatotoxicity. Notably, acute mortality occurred in both male and female groups treated with 3.0 mg k^−1^g DLin‐MC3‐DMA LNP, (4/5 deaths per group), indicating severe toxicity. Both SM‐102 and (*4S*)‐KEL12 LNPs exhibited more favorable safety profiles in the female groups receiving up to a dosage of 3.0 mg k^−1^g without a significant increase in ALT or AST levels. A gender difference was noted for SM‐102 LNP, as evidenced by significantly increased ALT and AST levels in males compared to females at a dosage of 3.0 mg k^−1^g. The ALT and AST levels in (*4S*)‐KEL12 LNP‐treated groups were significantly lower than those in SM‐102 LNP‐treated groups at a dosage of 3.0 mg k^−1^g for males, suggesting lower hepatotoxicity of (*4S*)‐KEL12 LNP. Consistently, histopathological analysis revealed liver necrosis in the DLin‐MC3‐DMA LNP group at a dosage of 3.0 mg k^−1^g, with no significant morphological changes in the liver or spleen for either SM‐102 or (*4S*)‐KEL12 LNP groups (Figure 5G). Taken together, we conclude that (*4S*)‐KEL12 LNP displayed superior safety profiles to those of SM‐102 or DLin‐MC3‐DMA LNP.

Additionally, we investigated the toxicity of (*4S*)‐KEL12 mRNA‐LNP after a single IM injection at dose levels of 75 and 225 µg mRNA per rat (n = 3–5/group). For ALT, neither the male nor the female group showed significant escalation at both dosages (Figure 5H,J). Interestingly, a reduction of ALT was observed in the male rats, especially in the (*4S*)‐KEL12‐LNP at the 225 ug dosage (Figure 5H). For AST, under both low and high dosages in both genders, no significant difference was detected between the control and (*4S*)‐KEL12 ‐LNP (Figure 5K), while DLin‐MC3‐DMA LNP showed higher AST than the control in all comparisons (Figure 5I). The higher sensitivity of the males to tested LNPs than the females was consistent with the IV results. To further investigate cumulative toxicologic effects, (*4S*)‐KEL12 mRNA‐LNP was administered once every week for 5 doses in Sprague‐Dawley rats (n = 10/group). No significant differences were detected in ALT, AST, creatinine (Cre), or total bilirubin (TBil) levels between the control and (*4S*)‐KEL12 mRNA‐LNP, suggesting a lack of cumulative toxicity (Figure 5L,M). We noted that SM‐102 and MC3 LNPs showed higher toxicity in male rats than in females. Similarly, Alnylam Pharmaceuticals reported higher sensitivity in male rats with their LNP candidate ALN‐VSP, yet a correlation with distribution was not established.^[^ ^9a^ ^]^ Therefore, we speculate that the increased toxicity of SM‐102 and MC3 in male rats might not be caused by different distribution.

### 2.7 Biodegradability of (4S)‐KEL12

**Figure 6:**
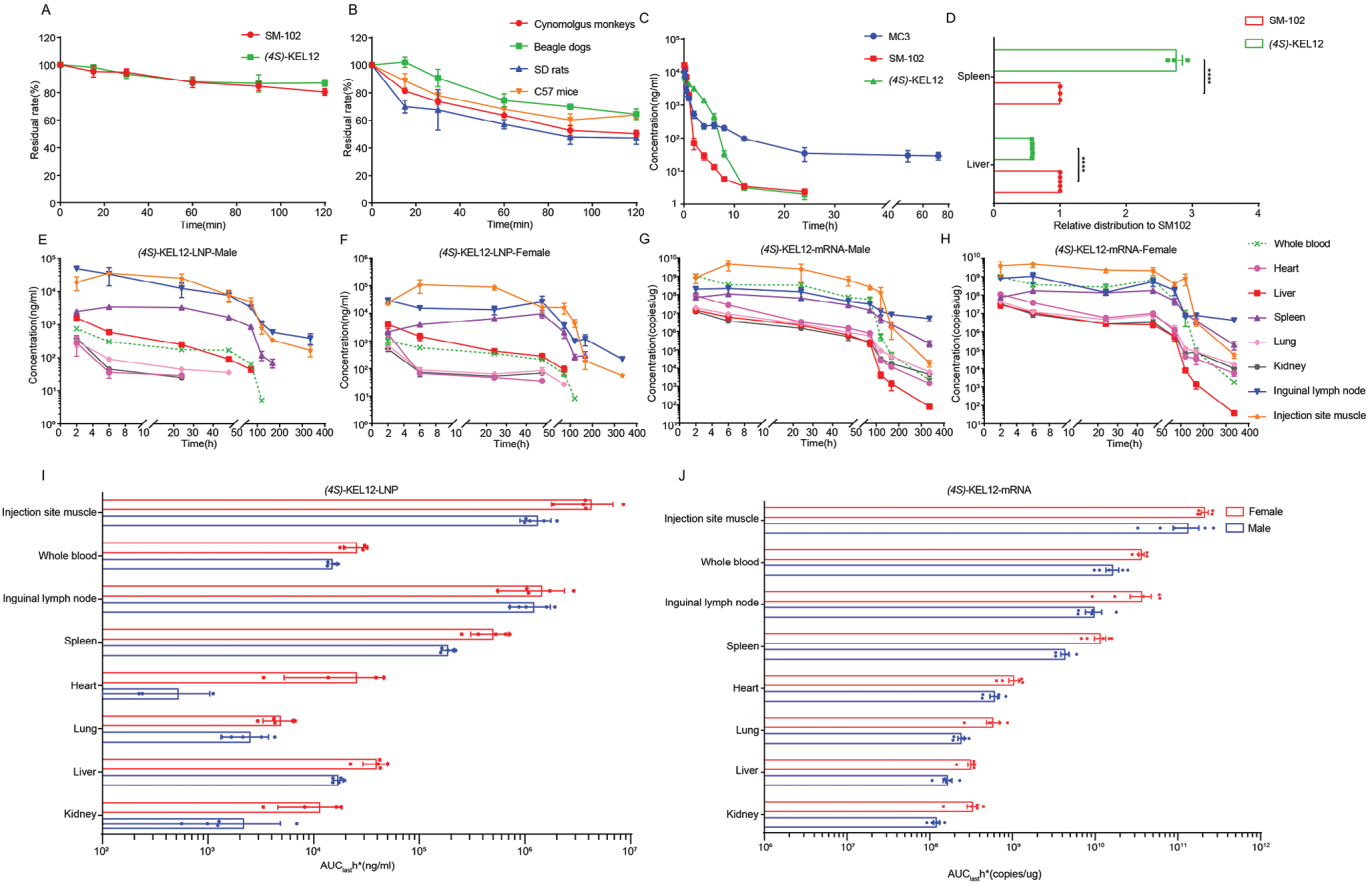
Biodegradability and distribution of (*4S*)‐KEL12 LNP. A‐B) In vitro liver microsomal stability. A) Relative remaining percentages of (red) SM‐102 and (green) (*4S*)‐KEL12 at various time points in human liver microsomes. B) Relative remaining percentages of (*4S*)‐KEL12 over time in liver microsomes across different species of Sprague‐Dawley rats (blue), C57 mice (orange), beagle dogs (green), and cynomolgus monkeys (red). C) Concentrations of different ionizable lipids in mouse plasma over time following IV injection of DLin‐MC3‐DMA (blue), SM‐102 (red), and (*4S*)‐KEL12 LNPs (green) (0.5 mg k^−1^g). D) Relative distribution of SM‐102 LNPmRNA (red) and (*4S*)‐KEL12 LNP mRNA (green) in the spleen and liver after IV injection. LNPs encapsulated with barcoded mRNA were IV injected into mice, followed by NGS of barcoded mRNAs in the liver and spleen. E‐F) Concentrations of ionizable lipid (*4S*)‐KEL12 over time in whole blood, organs, and tissues following IM injection. G‐H) Copies of (*4S*)‐KEL12 LNP mRNA over time in whole blood, organs, and tissues following IM injection. I‐J) The levels of ionizable lipid (*4S*)‐KEL12 (I) and mRNA (J) in whole blood, organs, and tissues following IM injection. Data were represented as mean ± SEM (n = 3–5). Statistical significance was calculated by unpaired one‐tailed Student's t‐test (D), **** *p* < 0.0001.

We first tested the in vitro biodegradability of the ionizable lipids by evaluating their liver microsomal stability. The levels of ionizable lipids were measured by LC‐MS/MS at different time points. As shown in **Figure** **6**A, about 80% of SM‐102 and 84% of (*4S*)‐KEL12 remained after 120 minutes, suggesting similar stability under the action of human liver microsomes. We then explored the stability of (*4S*)‐KEL12 across several mammalian species and detected significant differences. Specifically, (*4S*)‐KEL12 was degraded at a similar speed in cynomolgus monkeys and rats, with only 50.5% and 47.0% remaining after 120 minutes, respectively. Its degradation rate was slower in both C57 mice and beagle dogs, with 64.0% and 64.6% remaining after 120 minutes for each species (Figure 6B). These results indicated that (4S)‐KEL12 is biodegradable. Interestingly, Alnylam also reported that rats exhibited greater sensitivity to LNP toxicity compared to mice.^[^ ^9a^ ^]^ The difference in liver microsomal stability might be partially responsible for why rats are more sensitive to LNP toxicity.

We then conducted an in vivo biodegradation study of (*4S*)‐KEL12 LNP by measuring the levels of ionizable lipids in plasma following IV injection of *m*Luci LNPs (0.5 mg/Kg) in mice. During the first 6 hrs post‐injection, the levels of DLin‐MC3‐DMA or SM‐102 reduced faster than (*4S*)‐KEL12 (Figure 6C). This was consistent with a higher liver tropism of DLin‐MC3‐DMA and SM‐102 and the liver being the primary metabolizing organ, although other mechanisms likely also contribute. At later time points, (*4S*)‐KEL12 and SM‐102 maintained at relatively low levels (<10 ng/mL) after 12 hrs, and became undetectable after 24 hrs, whereas DLin‐MC3‐DMA LNPs remained at high levels at 12 and 24 hr time points. These results suggested that both (*4S*)‐KEL12 and SM‐102 exhibited good biodegradability.

### 2.8 Distribution of (4S)‐KEL12 LNP

To better understand the reduced liver expression of (*4S*)‐KEL12 LNP relative to SM‐102 LNP, we compared the biodistribution of the two LNPs in the liver and spleen. (*4S*)‐KEL12 and SM‐102 LNPs, each encapsulating a uniquely barcoded mRNA, were administered into mice at an equal molar ratio via IV, followed by tissue dissection and next‐generation sequencing (NGS) 4 hrs post‐injection. (*4S*)‐KEL12 LNP had significantly lower mRNA distribution in the liver and higher distribution in the spleen compared to SM‐102 LNP (Figure 6D). Decreased liver distribution was consistent with the reduced hepatic expression of (*4S*)‐KEL12 mRNA‐LNP (Figure 4G,H), as well as the improved safety profile of (*4S*)‐KEL12 LNP (Figure 5C,D).

To further characterize (*4S*)‐KEL12 mRNA‐LNP distribution in more detail, the levels of the (*4S*)‐KEL12 lipid and mRNA were measured by LC‐MS/MS and RT‐qPCR, respectively, using mouse blood and organs harvested at different times following IM injection. The peak times for mRNA and ionizable lipid levels were observed at 2 hours in most tissues, with the spleen and injection site muscle peaking at or after 6 hours (**Figure** **6**E‐H). The higher lipid levels observed in the liver, spleen, and lymph nodes, which were sustained for up to 72 hours, suggested active uptake. Tissues and organs with active uptake also showed higher expression, as demonstrated by the strong luciferase fluorescence observed in the liver and spleen in the LNP expression experiment (Figure 4). In contrast, the levels of the (*4S*)‐KEL12 ionizable lipid in the heart, lung, and kidney were consistently lower than in whole blood at all time points, suggesting that these organs received LNPs primarily through passive distribution via blood circulation rather than active uptake. After 120 hours, the (*4S*)‐KEL12 lipid was undetectable in major organs (heart, liver, lung, and kidney). This finding, along with its absence 24 hours after IV injection (Figure 6A), further confirms its good biodegradability.

The mRNA levels in the spleen and inguinal lymph nodes were significantly higher than those in the lung, heart, and kidney, consistent with the metabolic profile of (*4S*)‐KEL12 (Figure 6G,H,J). The mRNA levels in the liver were the lowest among all tissues, in agreement with the fact that the liver is a primary site for mRNA degradation. Furthermore, the AUCs (Area Under the Curve) of (*4S*)‐KEL12 and mRNA in the spleen were larger than those in the liver (Figure 6I,J), consistent with the barcode distribution results (Figure 6D). In addition, while our toxicological studies revealed no significant toxicity in either male or female rats treated with (*4S*)‐KEL12 LNP (Figure 5), we observed that mRNA and ionizable lipid levels were generally higher in female groups compared to male groups across various organs (Figure 6I,J).

### 2.9 Immunogenicity and Anti‐Tumor Effect of a Therapeutic mRNA (4S)‐KEL12‐LNP Vaccine

**Figure 7:**
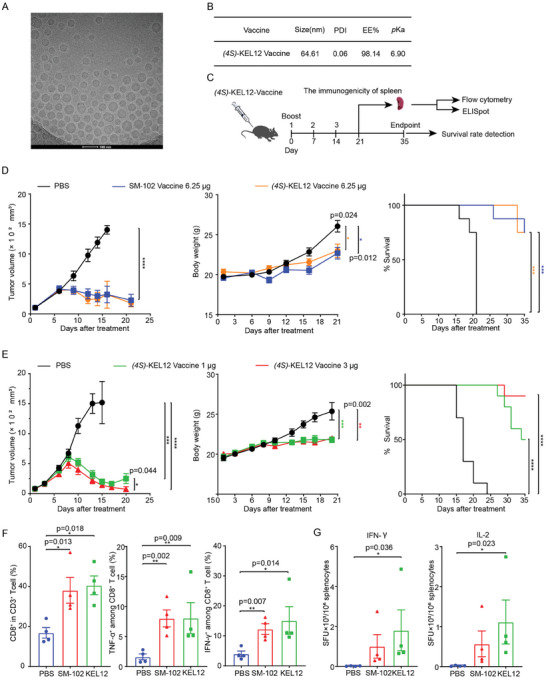
The therapeutic effect of (*4S*)‐KEL12 vaccine on the TC‐1 tumor model. A‐B) Cryo‐EM micrograph (A) and Physical properties (B) of (*4S*)‐KEL12 vaccine. Scale bar = 100 nM. C) Schematic representation of mice immunization schedule with (*4S*)‐KEL12 vaccine. D‐E) TC‐1 tumor‐bearing mice were immunized three times at one‐week intervals with specified doses. Tumor volume (Left), body weight (middle), and animal survival (right) were monitored and presented. PBS (black) treatment served as the negative control. D) Comparison of SM‐102 and (*4S*)‐KEL12 vaccine following IM administration at 6.25 µg. E) A dose de‐escalation study of (*4S*)‐KEL12 vaccine showing the impact of the vaccine at the dosage of (green) 1 ug or (red) 3 ug. F) CD8^+^, IFN‐γ, and TNF‐α positive cells were isolated by flow cytometry from the spleen of immunized mice at one week after three‐dose vaccines. G) The percentage of IFN‐γ and IL‐2 levels was determined by ELISpot assay in the spleen from immunized mice at one week after three‐dose vaccines. Mice immunized with PBS were used as negative controls. Data were presented as mean ± SEM (n = 4–15). Statistical significance was calculated by using multivariate analysis of variance (MANOVA) for (D and E) or One‐Way ANOVA test followed by Dunnett's post‐hoc test for (F and G). ns, *p* > 0.05; * *p* < 0.05; ** *p* < 0.01; *** *p* < 0.001.

To assess the potential advantages of (*4S*)‐KEL12 LNP in the development of mRNA‐based therapeutics, we evaluated the immunogenicity and anti‐tumor efficacy of (*4S*)‐KEL12 LNP encapsulating an mRNA encoding the E6/E7 antigens of HPV 16 and 18 as previously described.^[^ ^29^ ^]^ We formulated HPV E6/E7 mRNA using (*4S*)‐KEL12 LNP (hereafter (*4S*)‐KEL12 vaccine). Cryo‐electron microscopy (cryo‐EM) revealed that the (*4S*)‐KEL12‐based mRNA LNP displayed a spherical morphology with multi‐layered particles (**Figure** **7**A). (*4S*)‐KEL12 vaccine demonstrated high encapsulation efficacywith a similar *p*Ka value to that of *m*Luci (*4S*)‐KEL12 LNP (Figure 7B).

The anti‐tumor efficacy of the (*4S*)‐KEL12 vaccine was subsequently evaluated in the TC‐1 tumor model expressing HPV16 E6/E7 antigens. C57BL/6 mice subcutaneously implanted with TC‐1 tumor cells were intramuscularly administered with (*4S*)‐KEL12 vaccine on days 0, 7, and 14 after tumors reached an average volume of 100 mm^3^ (Figure 7C). (*4S*)‐KEL12 vaccine demonstrated comparable efficacy to the SM‐102 LNP encapsulated vaccine (SM‐102 vaccine hereafter) in tumor growth inhibition, and extending overall survival after immunization at a dose of 6.25 µg per mouse (Figure 7D). In a dose de‐escalation study, (*4S*)‐KEL12 vaccine further showed significant anti‐tumor efficacy at a dose as low as 1 µg per mouse (Figure 7E). It should be noted that the body weight trends for both the (4S)‐KEL12 and SM102 groups were lower than those of the PBS group (Figure 7D, middle). However, no significant difference in body weight was detected between the (4S)‐KEL12 and SM102 groups. The reduction in body weight in the vaccine‐treated groups is likely due to appetite suppression caused by the vaccines.

To understand the mechanistic insight of the observed anti‐tumor effect, we determined the immunogenicity of the (*4S*)‐KEL12 vaccine. Splenocytes from the 6.25 µg groups were collected one week after the last vaccination, followed by the analysis of cellular immunity. (*4S*)‐KEL12 vaccine significantly increased the frequency of antigen‐specific IFN‐γ^+^ or TNF‐α^+^ CD8^+^ T cells by intracellular cytokine staining (ICS) flow cytometry, indicating strong activation of antigen‐specific CD8^+^ T cell immunity (Figure 7F). Similarly, (*4S*)‐KEL12 vaccine resulted in a significant increase of IFN‐γ and IL‐2 by ELISpot (Figure 7G). The data were comparable between (*4S*)‐KEL12 vaccine and SM‐102 vaccine. These results together supported the potential application of (*4S*)‐KEL12 LNP in the development of mRNA‐based cancer vaccines.

## 3 Conclusion

Data from mechanistic studies and clinical trials demonstrated significant side effects and toxicity for commercially approved ionizable lipids, including DLin‐MC3‐DMA, ALC‐0315, and SM‐102 for RNA therapies.^[^ ^7^, ^9^, ^11^ ^]^ In this study, we hypothesized that the incorporation of multiple biodegradable groups within a single lipid could enhance safety profiles. We designed and synthesized a series of novel KELs incorporating a biodegradable ketal moiety in the linker and ester segments in the tails. Through iterative optimization of the head and tail groups for efficient spleen expression, we tuned the *p*Ka and molecular shapes of KELs, and identified (*4S*)‐KEL12 as a safe and efficient ionizable lipid for mRNA vaccine delivery. (*4S*)‐KEL12 LNP demonstrated a safety profile superior to both SM‐102‐ and DLin‐MC3‐DMA‐based LNPs, as evidenced by no mortality and lower ALT and AST levels at high dosage (3 mg k^−1^g, male rat). Additionally, (*4S*)‐KEL12 LNP exhibited an excellent immune organ tropism, with delivery efficacy comparable to SM‐102 in the spleen and significantly reduced expression in the liver. Detailed characterization further illustrated mRNA distribution patterns for the (*4S*)‐KEL12 and SM‐102 LNPs in the liver and spleen consistent with their different expression organ tropism. Finally, encapsulation of an mRNA encoding HPV antigens within (*4S*)‐KEL12 LNP resulted in robust cellular immune responses, leading to significant tumor regression and prolonged survival in tumor‐bearing mice. Overall, these results supported (*4S*)‐KEL12 LNP as a safe and efficacious delivery system for mRNA vaccines.

Although literature reported a number of ionizable lipids, the rational design of new lipids remains challenging. A combinatorial chemistry‐based approach has been employed to identify novel lipids.^[^ ^14^, ^30^ ^]^ A key feature of this approach was the development of a one‐step synthetic strategy that allowed the rapid generation of a lipid library.^[^ ^31^ ^]^ Here we instead adopted medicinal chemistry approaches, guided by the putative in vivo mechanism of ionizable lipids for rational lipid design. Specifically, we combined the biodegradable ester and ketal moieties into the same lipid molecule to reduce toxicity. Iterative optimization was performed on both the head group and tail group to fine‐tune their *p*Ka values and molecular shapes for enhanced delivery efficiency. Through synthesis of a limited number of compounds, we successfully identified (*4S*)‐KEL12 with potent delivery activity and reduced hepatotoxicity.

Our detailed characterization demonstrated a reduced liver tropism and a relatively better spleen tropism for (*4S*)‐KEL12 LNP than for SM‐102 LNP using comprehensive approaches. Specifically, (*4S*)‐KEL12 LNP afforded a luciferase reporter activity similar to SM‐102 LNP in the spleen, but much lower than SM‐102 LNP in the liver (Figure 4E,F). We provided corroborating mechanisms about the observed organ tropism from several aspects. The different organ tropism was at least partially explained by the differential mRNA distributions in the spleen and liver for the two LNPs (Figure 6D). Moreover, pharmacokinetic data for KEL‐12 LNP suggested a greater distribution of both lipid and mRNA in the spleen than in the liver (Figure 6I,J). The serum incubation experiment suggested a possible role of the protein corona (Figure S4). Reduced expression of KEL12 LNP in liver endothelial cells and Kupper cells compared to SM‐102 LNP provided a more detailed account of the limited liver tropism after IM delivery (Figure S3). Interestingly, the two LNPs showed quite different expression trends in macrophages and DCs over time in the spleen and lymph nodes. Further characterization confirmed that KEL12 LNP is at least comparable to SM‐102 LNP in protein expression in several immune cell types, with significantly higher efficiency than SM‐102 LNP in DCs in both spleen and inguinal lymph nodes (Figure 4I,J). Consistently, the (*4S*)‐KEL12 vaccine displayed similar activity to the SM‐102 vaccine in immunogenicity and anti‐tumor efficacy by IM administration. These results together suggested that it is critical to evaluate LNP delivery efficacy in immune organs and cells for mRNA vaccine development.

Both (*4S*)‐KEL12 and SM‐102 possess biodegradable ester moieties in their tails. Hence, it is reasonable that (*4S*)‐KEL12 LNP and SM‐102 LNP displayed better biocompatibility and lower toxicity than DLin‐MC3‐DMA LNP. (*4S*)‐KEL12 LNP showed significantly lower liver tropism than SM‐102 LNP, which may partially explain its reduced hepatotoxicity. Additionally, (*4S*)‐KEL12 demonstrated good biodegradability, as it was undetectable in major organs (heart, liver, lung, kidney) 120 hours after IM injection and undetectable in the blood 24 hours after IV injection.

Recently, Dahlman et al. demonstrated the influence of stereochemistry in 20‐hydroxycholesterol on mRNA LNP delivery efficiency.^[^ ^28^ ^]^ Despite the presence of chiral carbons in many reported lipids such as ALC‐0315 and ckk‐E12, there is a lack of literature studying the relationship between delivery efficacy and stereochemistry of ionizable amino lipids. Our results showed no significant difference in delivery efficacy (Figures 3 and S1), as well as the efficacy in an mRNA HPV therapeutic vaccine (Figure S5), indicating no universal relationship between delivery efficacy and stereochemistry for delivery lipids.

In summary, this study identified (*4S*)‐KEL12 as a safe, biodegradable, and efficient ionizable lipid for mRNA vaccine delivery. We presented comprehensive data encompassing pharmacokinetics, distribution, biodegradability, and expression at organ and/or cell type levels to understand the favorable immune cell tropism and reduced hepatotoxicity of (4S)‐KEL12 LNP. Encapsulation of an mRNA encoding the HPV antigens within (*4S*)‐KEL12 LNP resulted in robust efficacy in cancer therapy in an animal model. The remarkable therapeutic efficacy, along with its favorable safety profile, suggests that (*4S*)‐KEL12 LNP holds great promise for future clinical applications. The multi‐dimensional analysis of the SAR, toxicity, biodegradability, distribution, and stereochemistry of the lipids presented in this study will greatly contribute to the rational design and discovery of novel lipid‐based delivery systems.

## 4 Experiment Section

### Materials

SM‐102, DLin‐MC3‐DMA, and DMG‐PEG 2000 were purchased from SINOPEG. Chol and DSPC were purchased from Nippon Fine Chemical. All commercially available solvents and reagents were used without further purification. The reagents were all of analytical grade or chemically pure. *Fluc m*RNA and hEPO mRNA were prepared in RinuaGene Biotechnology, Co.

### Animals

Balb/c mice (6‐8 weeks old, 18–22 g), Sprague‐Dawley rats (6‐8 weeks, 180–200 g), and C57BL/6 mice (6‐8 weeks old, 18–22 g) were purchased from Vital River. The *C57BL/6Smoc‐Gt(ROSA)26Sor^em(CAG‐LSL‐tdTomato)1Smoc^* mice expressing a CAG‐LSL‐tdTomato transgene that can be activated by the CRE recombinase were provided by GenoBioTX (NM‐KI‐225042, GenoBioTX). The animals were kept under standard and pathogen‐free conditions (25 °C, 50 ± 10% humidity, 12‐hour dark/light cycle) with free access to food and water. Animal studies were conducted in accordance with the guidelines of the Chinese Association for Laboratory Animal Sciences and approved by the IACUC of Ascentage Pharma (Approved ethical number: AS‐20230621‐01, AS‐20240313‐01, AS‐20221228‐01).

### Cell Lines

The Human Embryonic Kidney 293T cell line and the Hepa 1–6 liver cancer cell line were purchased from the Stem Cell Bank, Chinese Academy of Sciences, and ATCC, respectively. HEK293T and Hepa 1–6 cell lines were cultured in Dulbecco's minimal essential medium (Gibco) supplemented with 10% fetal bovine serum (FBS) (Gibco) and 1% penicillin/streptomycin (Gibco). TC‐1 tumor cells transformed with HPV16 E6/E7 and c‐Ha‐ras oncogenes were kindly provided by Professor Mingzhao Zhu (Institute of Biophysics, Chinese Academy of Sciences, Beijing, China). TC‐1 tumor cells cells were maintained in RPMI‐1640 media (Gibco) supplemented with 10% FBS.

### Synthesis and Characterization of KELs

The synthetic procedures of the KELs were given in the supporting information. All KELs were characterized with ^1^H NMR, ^13^C NMR, and ESI‐MS.

### LNP Formulation and Characterization

Lipid components (50% ionizable lipid, 38.5% Chol, 10% DSPC, 1.5% PEG‐DMG) were dissolved in ethanol, and RNA was dissolved in 20 mM sodium acetate buffer (pH 5.0). LNPs were prepared by mixing the organic phase and the aqueous phase in the T‐mixer at a total flow rate of 12 mL mi^−1^n (aqueous/organic flow rate ratio of 3). Formulated LNPs were diluted with an equal volume of 500 mM sucrose and collected in an ultrafiltration centrifugal tube with 100 kDa NMWCO. LNPs were concentrated, and buffer exchanged with an equal volume of 2 mM acetate 250 mM sucrose buffer for 5 times. Tromethamine buffer was added to the LNPs to adjust the LNP pH to 7.5. LNPs were sterile filtered using a 0.22 µm polyethersulfone syringe filter (Cobetter, Uniflo 33), and stored at ‐20 °C prior to use.

### LNP Size and Distribution and Encapsulation Efficiency

The sizes of the LNP formulations were assessed by DLS (Malvern Zetasizer Pro). Quant‐it RiboGreen assay (ThermoFisher Scientific, Cat# R11490) was used to measure the encapsulation efficiency of the LNP formulations. Briefly, LNPs treated by 1% (v/v) Triton X‐100 (Adamas‐beta) to disrupt LNP structure and release mRNA and untreated LNPs were diluted to a concentration below 2 µg mRNA/mL, then reacted with an equal volume of RiboGreen assay solution at a 200‐fold dilution. Standard curves were generated within the range between 0.0 to 2.0 µg mRNA/mL using a series of free mRNA solutions with or without 1% v/v Triton X‐100. The concentrations of free mRNA and total mRNA in the formulation were determined using bulk fluorescent reading (excitation: 490 nm, emission: 535 nm) of the sample against the corresponding standard curves. The amount of RNA loaded into the LNPs (internal RNA) was calculated by subtracting the values obtained in 1 x TE (external RNA) from 1 x TE + 1% Triton X‐100 (total RNA). Subsequently, the encapsulation efficiency was calculated as the percentage obtained by dividing the amount of internal RNA by the total RNA amount.

### LNP Apparent pKa

A series of buffers with pH values varying by 1 between 3.0 and 12.0 were prepared by titrating a solution containing 10 mM citrate, 10 mM phosphate, 10 mM borate, and 150 mM NaCl with 1.0 M HCl and 0.5 M NaOH. LNPs were diluted to 75 µM ionizable lipid, TNS to 6 µM in these buffers. Fluorescence intensity was determined using a Thermo Scientific 3020 spectrophotometer (325 nm excitation/435 nm emission). With the resulting fluorescence values, a sigmoidal plot of fluorescence versus buffer pH was created. The log of the inflection point of this curve was the apparent *p*Ka of the LNP formulation.

### In vivo Firefly Luciferase mRNA Expression

Balb/c mice were injected intramuscularly (0.25 mg k^−1^g) or intravenously (1 mg k^−1^g) with LNPs. At 6 or 24 hrs post‐administration, mice were injected intraperitoneally with 150 mg k^−1^g of *D‐*luciferin monosodium salt (ThermoFisher) and imaged using the in vivo imaging system (IVIS Lumina LT Series III) 10 min post intraperitoneally. 1 × PBS (IM: 50 µL; IV: 200 µL) was injected as a negative control.

### In vivo hEPO mRNA Expression

Balb/c mice were injected intramuscularly or intravenously (5 µg/mouse) with LNPs encapsulating *hEPO* mRNA. Blood was collected from mice via the orbital and allowed to clot at room temperature in blank tubes. The tubes were then centrifuged at 3,000 g for 10 min, and the serum samples were aliquoted and stored at ‐20 °C until analysis. hEPO concentrations were determined using a Human EPO ELISA Kit (catalog #PE230; Beyotime Biotech Inc) according to the manufacturer's instructions.

### Flow Cytometry Analysis of tdTomato Expression upon CRE Recombinase mRNA‐LNP Delivery in Liver Cell Types of the C57BL/6Smoc‐Gt(ROSA)26Sor^em(CAG‐LSL‐tdTomato)1Smoc^ Transgenic Mouse Model

For flow cytometry analysis on single cell suspension, LNPs encapsulating mCre mRNA at a dose of 0.5 mg k^−1^g were IM administered to the transgenic mice (GenoBioTX). After 3 days, mice were sacrificed, and livers were cut into small pieces before digestion with hyaluronidase (Absin, abs47014926) and collagenase (Absin, abs47048003) in RPMI‐1640 (Sigma, R8758‐500ML) at 37 °C. Cells were harvested by centrifugation at 1,500 rpm for 5 min under 4 °C, then resuspended in red blood cell lysis buffer (Solarbio, R1010). After incubation, 3 mL of 1x complete RPMI‐1640 media with 10% FBS was added to stop red blood cell lysis. Cells were centrifuged again for 5 min before resuspension. Cells were fixed and stained with fluorochrome‐conjugated monoclonal antibodies for cell surface markers. Liver endothelial cells were identified as CD45^−^CD31^+^, and Kupper cells were identified as CD45^+^CD31^−^F4/80. Antibodies used for flow cytometry included PerCP‐Cy5.5 Rat Anti‐Mouse CD45(30‐F11) (#550 994, BD Pharmingen), BD OptiBuild BV605 Rat Anti‐Mouse CD31 (#740 356, BD Pharmingen), APC Rat Anti‐CD11b(M1/70) (#553 312, BD Pharmingen), and BD Horizon BV421 Rat Anti‐Mouse F4/80 (#565 411, BD Pharmingen). The stained cells were washed once with PBS before flow cytometry using FlowJo v10 software.

### In vitro Serum Incubation, Transfection, and Firefly Luciferase Activity Experiment

Hepa1‐6 cells were seeded at a density of 3 × 10^5^ cells/mL in a 12‐well plate and were cultured for 16–20 h before mRNA‐LNP transfection. *mLuc* mRNA were encapsulated into MC3, SM102, and (4S)‐KEL12 LNP, respectively, then incubated with freshly prepared mouse serum of BALB/c at an LNP: serum ratio of 1: 2 (v/v) at 37 °C for 30 min. Subsequently, 1 µg of serum‐treated or non‐treated *mLuc* mRNA LNP was added into Hepa 1–6 cells. Luciferase activity was determined 6 h after LNP transfection using the Luciferase Assay System kit (Cat# E1501, Promega). The bioluminescence signals were collected using Centro Microplate Luminometer (Bethold).

### Isolation and Identification of Plasma Proteins Bound to LNPs by LC‐MS/MS

100 µL of LNPs of SM102 or (*4S*)‐KEL12 were incubated with 100 µL of freshly‐prepared mouse serum (Balb/C, 8‐weeks old, female) at 37 °C for 30 min. LNPs were then purified using Spin‐columns for Liposome Purification (ProFoldin, #SLP20) and before LC‐MS analysis. After reduction‐alkylation followed by tryptic enzymolysis, protein samples were analyzed using liquid chromatography coupled with tandem mass spectrometry (LC‐MS/MS) at the Shanghai Majorbio Bio‐Pharm Technology Co., Ltd. (Shanghai, China).

### Cell Counting Kit‐8 Assays

293T Cells were maintained in DMEM (high glucose) supplemented with 10% (v/v) fetal bovine serum at 37 °C in 5% CO_2_ environment. Briefly, cells (2, 000 per well) in DMEM (100 µL) were seeded overnight in 96‐well plates, then incubated with ionizable lipids at varying concentrations (0, 0.3, 1, 3, 10, 30, 100 µM for both components) or luciferase mRNA LNP in triplicates at the concentrations of 200 ng, 100 ng, and 50 ng per well, respectively. After 48 h incubation, 10 µL CCK8 was added and incubated for 2 h, and the absorbance was recorded at 450 nm using a microplate reader (BioTek, Synergy LX).

### In vivo Toxicity Evaluation

IV toxicity: *(4S)*‐KEL12 LNP encapsulated with luciferase mRNA was administered at 0.3, 1, or 3 mg k^−1^g (5 Sprague‐Dawley rats /group/gender) via single intravenous bolus injection. 1.0 ml of blood was obtained 24 hours post‐dose from the jugular vein of conscious animals using manual restraint and was processed into serum. Potential test article‐related effects were evaluated by the assessment of clinical signs and serum chemistry (complete panel,7060 Automatic Analyzer (Hitachi)).

IM toxicity: *(4S)*‐KEL12 LNP encapsulated with E6/E7 antigens of HPV 16 and 18 was administered at 75 and 225 µg/rat (3‐5 Sprague‐Dawley rats/group/gender) via single IM injection. 1.0 ml of blood was obtained 24 hours post‐dose from the jugular vein of conscious animals using manual restraint and was processed to serum. Potential test article‐related effects were evaluated by the assessment of clinical signs and serum chemistry (complete panel,7060 Automatic Analyzer (Hitachi)).

Cumulative toxicity after IM: (*4S*)‐KEL12 LNP encapsulated with E6/E7 antigens of HPV 16 and 18 was administered once every week for 5 doses in Sprague‐Dawley rats (on day 1, day 8, day 15, day 22, and day 29, n = 10/group). Biochemical parameters were then tested on day 32.

### Organ Tropism Assay

The *m*Luci LNP was injected into the Balb/c mice through IV (1 mg k^−1^g) or IM (0.25 mg k^−1^g) injection, mice were injected intraperitoneally with 150 mg k^−1^g of *D‐*luciferin monosodium salt (ThermoFisher). 10 mins later, mice were sacrificed, and the main organs (heart, liver, lung, spleen, and kidney) were collected and imaged using the in vivo imaging system (IVIS Lumina LT Series III).

### Biodegradability of LNPs after IV Injection

Balb/c mice (n = 9) were intravenously (0.5 mg k^−1^g) injected on with mLuci‐LNPs. Mouse blood was collected from three mice at each time point: 5 min, 15 min, 30 min, 1 h, 2 h, 4 h, 6 h, 8 h, 12 h, 24 h, 48 h, and 72 h. All blood samples were centrifuged at 5000 r/min for 5 min to obtain serum which was then stored at −20 °C. 30 µL of the serum was added to 90 µL of ethanol containing internal standard and the mixture was centrifuged at 12 000 r/min for 10 min to remove protein. All specimens were quantified by the LC‐MS/MS.

### Microsomal Stability Assay of Ionizable Lipids

To determine the microsomal stability, microsomes were preincubated with ionizable lipids for 10 min at 37 °C in potassium phosphate buffer (50 mM, pH 7.4). The final incubation mixtures consisted of 0.5 mg of microsomal protein/mL liver microsomes, 5 mM MgCl_2_, and 0.0025 mg/mL alamethicin in potassium phosphate buffer (50 mM, pH 7.4). The reactions were initiated with the addition of NADPH and UDPGA regenerating system. At different time points (0, 15, 30, 60, 90, and 120 min), an aliquot (50 µL) sample was taken and quenched with 200 µL of acetonitrile containing internal standard. Following precipitation and centrifugation, the supernatants were analyzed by LC‐MS/MS. This study was conducted at Safe Medical Tech Co. Ltd (Langfang, Hebei China).

### Liver and Spleen Distribution of LNPs Encapsulated with Barcoded mRNA

Barcoded mRNA was prepared as previously described.^[^ ^32^ ^]^ Briefly, DNA templates were obtained by PCR using a forward primer and reverse primers containing different barcodes for each lipid, UMI (unique molecular identifier) of a 12 nt oligonucleotide, DOCK (5′‐CTATTCGGCTATGACTGGGC‐3′), followed by purification of PCR products using Macherey‐Nagel^TM^ Gel and Nucleospin PCR Clean‐up (Takara Bio, Cat#740609.250). The barcodes 5′‐CCGCGGTT‐3′ and 5′‐GGACTTGG‐3′ were used for labeling SM102 and (4S)‐KEL12, respectively. Barcoded mRNA was synthesized by in vitro transcription and encapsulated into LNP of SM102 and (4S)‐KEL12. To study the in vivo biodistribution, LNP mixture (SM102‐LNP: (4S)‐KEL12‐LNP = 1:1) was injected into female BalB/c mice. Liver and spleen were collected at 4 h after injection, followed by mRNA preparation using VAHTS mRNA Capture Beads (Vazyme, Cat# N401) and library construction using Phusion High Fidelity DNA Polymerase (NEB, cat# M0530S) and VAHTS DNA Clean beads (Vazyme, cat# N411‐01). NGS were performed at Novogen (Tianjin, China) using Illumina MiSeq (Illumina), and the generated data were analyzed following the reported method.^[^ ^32^ ^]^

### Distribution Study of (4S)‐KEL12 LNP

*(4S)*‐KEL12 LNP encapsulated with E6/E7 antigens of HPV 16 and 18 was administered at 75 µg/mouse (40 female and 40 male) via single IM injection. Mouse blood was collected, and organs and tissues were harvested at 2 h, 6 h, 24 h, 48 h, 72 h, 120 h, 168 h, and 336 h. The mRNA levels were quantified using RT‐qPCR, and the levels of ionizable lipid were measured using LC‐MS/MS.

### Antitumor Activity of (4S)‐KEL12 Vaccine in Mouse TC‐1 Tumor Model

The murine TC‐1 tumor cell line, which was derived from primary lung epithelial cells, immortalized with HPV16 E6/E7 and transformed with c‐Ha‐ras oncogene.^[^ ^33^ ^]^ For therapeutic tumor experiments, TC‐1 cells were harvested during the exponential growth phase. Female C57BL/6 mice were injected with 5 × 10^5^ TC‐1 tumor cells subcutaneously. When the tumors reached an average of 80–110 mm^3^, the vaccine was intramuscularly administrated three times at one‐week intervals unless otherwise specified. Tumor sizes were measured using an electronic caliper every two to three days for calculating tumor volumes using the equation (width × width × length)/2. Animals were euthanized when either mouse showed signs of suffering or the average tumor volume exceeded 2000 mm^3^.

### Immunogenicity

One week after the third vaccination, spleens were isolated and suspended in 2 mL RPMI‐1640 medium containing 10% fetal bovine serum (FBS). Splenocytes were stimulated with peptide pool (2 µg/mL/peptide), or medium only for ELISpot, and with 10 µg/mL brefeldin A (#B8581, Solarbio) for flow cytometry with a 20 h incubation at 37 °C. Peptides were synthesized by SBS Genetech. The ELISpot assay was conducted using the mouse IFN‐γ (#2 110 002, Dakewe) and IL‐2 (#3441‐4APW‐2, Mabtech) ELISPOT kit according to the manufacturer's instructurions. The spots of each mouse were analyzed by the Mabtech IRIS FluoroSpot/ELISpot reader.

### Flow Cytometry

Flow cytometry analysis was performed on the single cell suspension of splenocytes. Monoclonal antibodies against CD3, CD8α, CD4, CD44, CD62L, and CD25 for extracellular staining and antibodies against IFN‐γ, IL‐2, granzyme B, and Foxp3 for intracellular staining were purchased from BioLegend. The stimulated single cells were stained for extracellular targets at 4 °C for 30 min after viability dyes and blocking using anti‐mouse CD16/32 antibody (Biolegend). After washing, the cells were fixed and permeabilized at 4 °C for 30 min using the Foxp3 transcription factor staining buffer set according to the manufacturer's instructions (eBioscience). Intracellular IFN‐γ, IL‐2, granzyme B, and Foxp3 were stained at 4 °Cfor 1 h. After washing, the cells were resuspended in PBS and analyzed for flow cytometry using a CytoFLEX flow cytometer (Beckman Coulter).

### Statistical Analysis

Data from at least three independent experiments were presented as mean ± SEM. Unpaired one‐sided t‐tests, one‐way ANOVA, or MANOVA with an appropriate post‐hoc all pairwise multiple comparison test were used to determine significance as indicated in the figure legends. The number of replicates is also detailed in the figure legends. Differences were considered statistically significant when *p* < 0.05. Statistical analyses were performed using GraphPad Prism 8 software.

## Conflict of Interest

The authors declare no conflict of interest.

## Ethics statement

All mice were maintained under SPF conditions, and animal experiments were approved by the Institutional Animal Care and Use Committee (IACUC) of Ascentage Pharma (Approved ethical number: AS‐20230621‐01, AS‐20240313‐01, AS‐20221228‐01).

## Supporting information



Supporting Information

Supplemental Dataset 1

## Data Availability

The data that support the findings of this study are available from the corresponding author upon reasonable request.
