# Supplementary material for: Discovery of Ketal‐Ester Ionizable Lipid Nanoparticle with Reduced Hepatotoxicity, Enhanced Spleen Tropism for mRNA Vaccine Delivery
Source: Adv Sci (Weinh). 2024 Oct 10;11(45):2404684. doi: 10.1002/advs.202404684 (PMC11615764; doi:10.1002/advs.202404684)
Supplement: Supplementary file 1 — Supporting Information [file ADVS-11-2404684-s002.docx]

**Supplementary information**

**Discovery of ketal ester ionizable lipid nanoparticle with reduced hepatoxicity and enhanced spleen tropism for mRNA vaccine delivery**

1. Supplementary figures S1-5
2. Experimental section of chemistry
   1. Synthesis of KEL lipids
   2. Purity determination and chiral separation
3. **Supplementary figures**

**
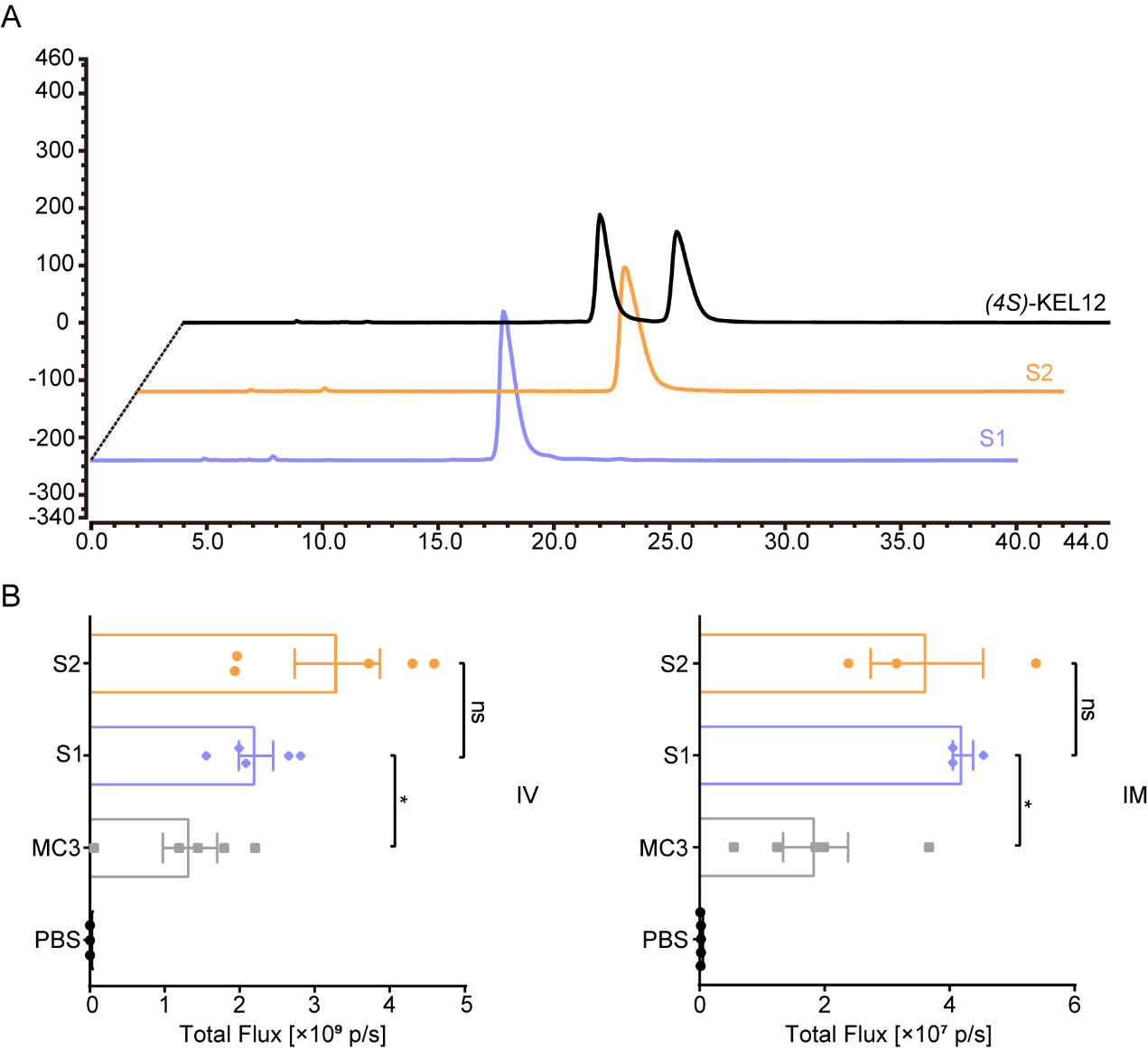
**

**Figure S1.** The impact of stereochemistry on the delivery efficacy. A) HPLC spectra of *(4S)*-KEL12, S1 and S2. B) Total luminescent flux in the whole body of female Balb/C mice at 24 h post-injection of *mLuci* LNP. Data are presented as mean ± SEM. Error bar = SEM.

**
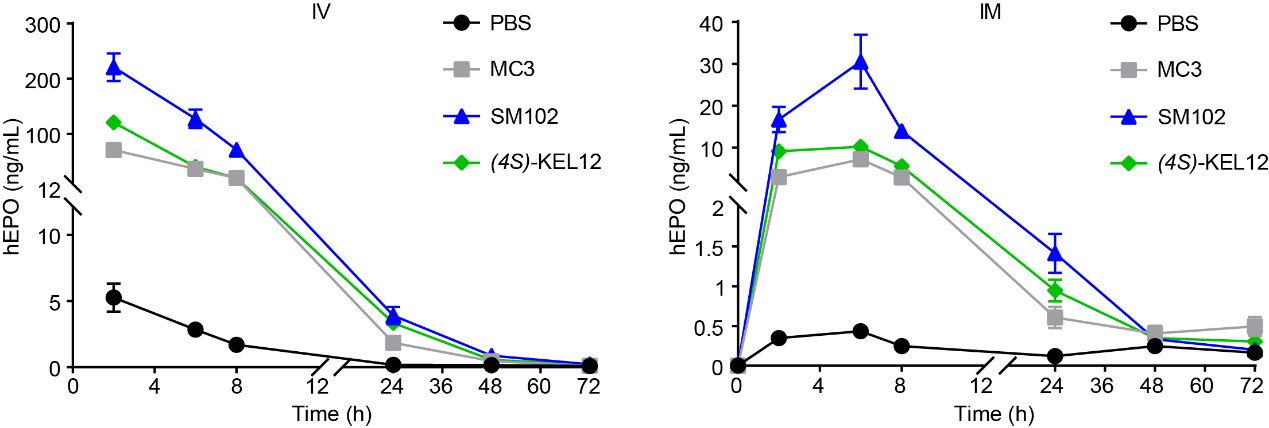
**

**Figure S2.** hEPO plasma concentrations. hEPO plasma concentrations of female Balb/C mice after IV or IM delivery of 0.25 mg/kg *hEPO* mRNA formulated in different LNPs (n=5). Data are presented as mean ± SEM; error bar = SEM.


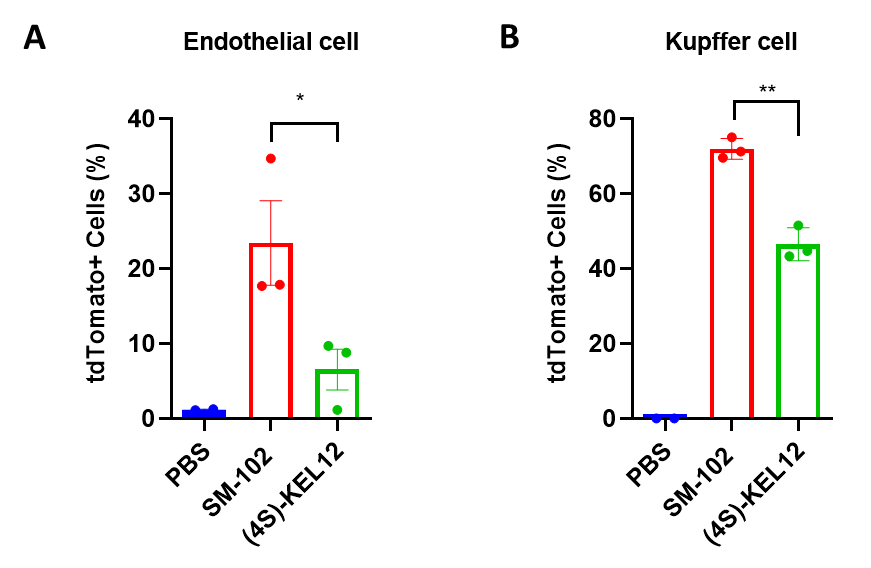


**Figure S3.** tdTomato expression after *in vivo* delivery of Cre recombinase mRNA by KEL12 LNP or SM-102 LNP into *Ai14::tdTomato-stop* mice by IM injection (n=3). PBS injection served as the negative control. Ratios of tdTomato^+^ cells in both (A) endothelial cells and (B) Kupffer cells from the liver were lower for KEL12 LNP than for SM-102 LNP 72hours after IM injection measured by FACS. *, p<0.05; **, p<0.01. Student’s t-test.


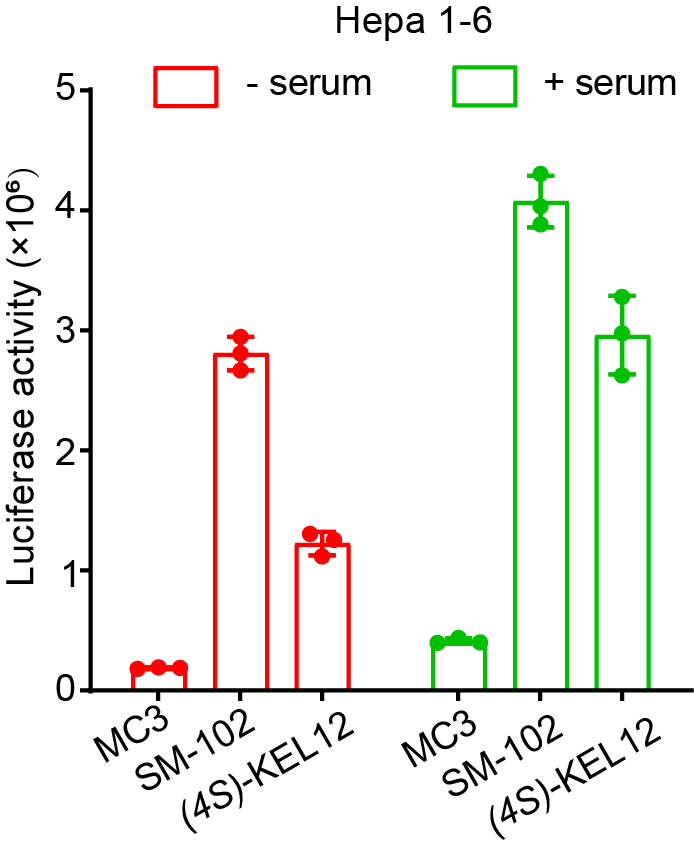


**Figure S4.** Luciferase activity 6 hrs after transfection of different *m*Luci LNPs into Hepa 1-6 liver cancer cells with (+serum, green) or without (-serum, red) incubation with mouse plasma. MC3 LNP, SM-102 LNP and KEL12 LNP were incubated with mouse plasma at 37°C for 30 min before luciferase activity assay. Cells transfected by KEL12 LNP exhibited luciferase activity lower than SM102 LNP but higher than MC3 LNP regardless of plasma incubation.

**
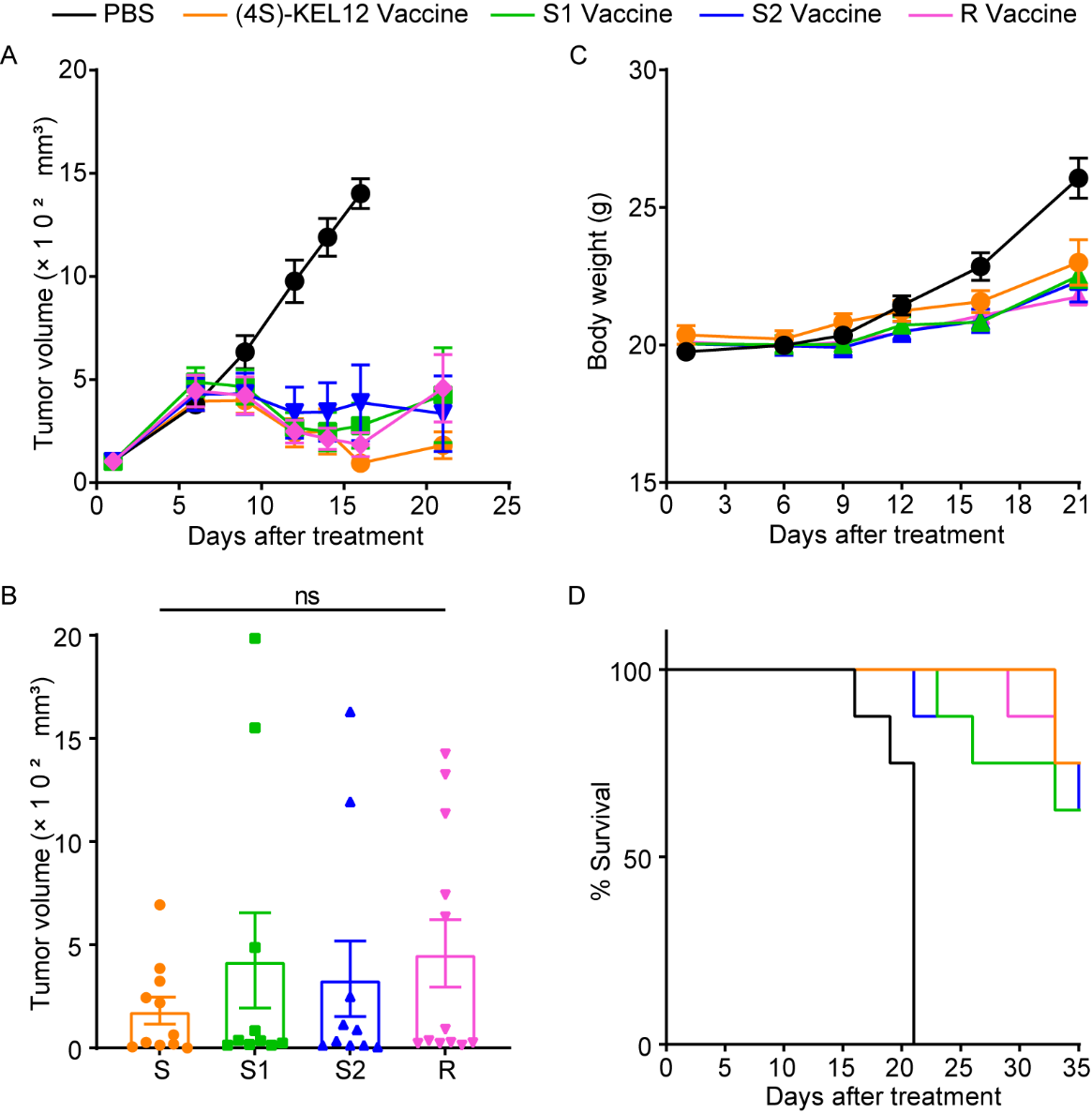
**

**Figure S5.** The therapeutic effect of KEL12’s isomers vaccine on the TC-1 tumor model.

**2．Experimental section of chemistry**

**2.1. Synthesis of KEL lipids:**


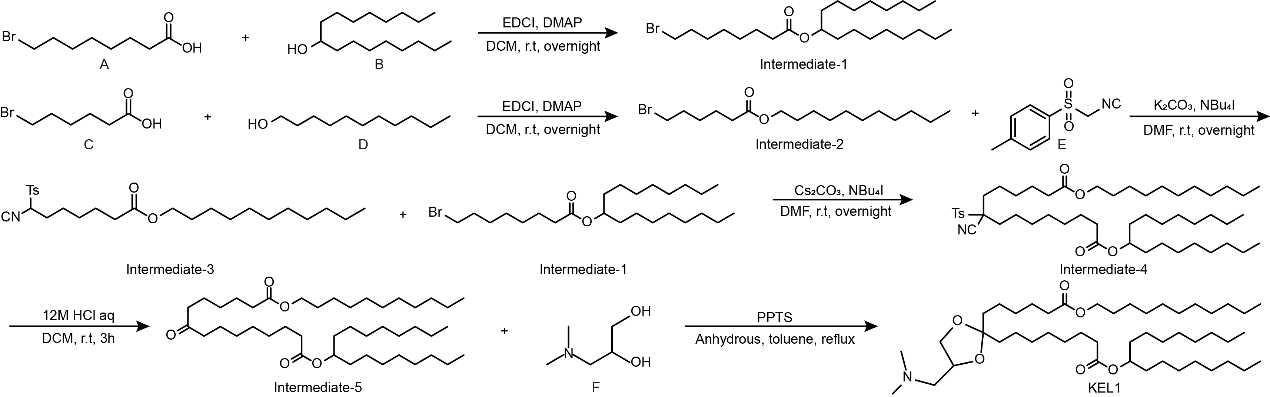


**Scheme S1.** Synthesis of KEL1.

**Synthesis of KEL1:** Intermediate-1: To an oven dried 250 mL round-bottom flask equipped with a magnetic stir bar taken A (8-Bromooctanoic acid, 10.5 g, 46.8 mmol) and then suspended in 100 mL of DCM under argon atmosphere. N-(3-Dimethylaminopropyl)-N′-ethylcarbodiimide hydrochloride (EDCI, 11.6 g, 60.8 mmol) and 4-Dimethylaminopyridine (DMAP, 1.9 g, 18.7 mmol) were then added and allowed to stir for 15 min. B (9-Heptadecanol, 10.0 g, 39.0 mmol) was then added and allowed to stir at room temperature. After the reaction was completed, an equal volume of saturated sodium bicarbonate solution was added to the reaction solution to dilute and separate, and anhydrous magnesium sulfate was added to the organic layer to dry, filter, and solvents were removed in vacuo. The product was purified by SiO_2_ gel rapid column chromatography. Petroleum ether and ethyl acetate were used as eluents at 30:1 to obtain intermediate 1, which was colorless oily liquid. Yield: 74%, 16.02 g.

Intermediate-2: To an oven dried 250 mL round-bottom flask equipped with a magnetic stir bar taken C (6-Bromohexanoic acid, 9.6 g, 49.0 mmol) and then suspended in 100 mL of DCM under argon atmosphere. EDCI (10.2 g, 53.1 mmol) and DMAP (2.0 g, 16.4 mmol) were then added and allowed to stir for 15 min. D (1-Undecanol, 7.0 g, 40.8 mmol) was then added and allowed to stir at room temperature for overnight. After the reaction was completed, an equal volume of saturated sodium bicarbonate solution was added to the reaction solution to dilute and separate, and anhydrous magnesium sulfate was added to the organic layer to dry, filter, and solvents were removed in vacuo. The product was purified by SiO_2_ gel rapid column chromatography. Petroleum ether and ethyl acetate were used as eluents at 30:1 to obtain intermediate 2, which was colorless oily liquid. Yield: 86%, 12.20 g.

Intermediate-3: To an oven dried 250 mL round-bottom flask equipped with a magnetic stir bar taken Intermediate-2 (10.0 g, 28.7 mmol) and then suspended in 100mL of DMF under argon atmosphere. E (4-(Methylphenyl) sulfonylacetonitrile, 13.7 g, 70.28 mmol), K_2_CO_3_ (14.6 g, 105.4 mmol) and NBu_4_I (13.0 g, 35.1 mmol) were then added and allowed to stir at room temperature for 12 h. The reaction was monitored by TLC. After the reaction was completed, an equal volume of water was added to the reaction solution, diluted, extracted with ethyl acetate (50 ml × 3), and solvents were removed in vacuo. The product was purified by SiO_2_ gel rapid column chromatography. Petroleum ether and ethyl acetate were used as eluents at 15:1 to obtain intermediate 3, which was white solid. Yield: 83%, 11.20 g.

Intermediate-4: To an oven dried 250 mL round-bottom flask equipped with a magnetic stir bar taken Intermediate-3 (11.2 g, 23.70 mmol) and then suspended in 100 mL of DMF under argon atmosphere. Intermediate-1 (16.02 g, 35.60 mmol), Cs_2_CO_3_ (15.4 g, 47.43 mmol) and NBu_4_I (13.1 g, 35.60 mmol) were then added and allowed to stir at room temperature for 12 h. The reaction was monitored by TLC. After the reaction was completed, an equal volume of water was added to the reaction solution, diluted, extracted with ethyl acetate (50 ml × 3), and solvents were removed in vacuo. The product was purified by SiO_2_ gel rapid column chromatography. Petroleum ether and ethyl acetate were used as eluents at 3:1 to obtain intermediate 4, which was colorless oily liquid. Yield: 88%, 17.60 g.

Intermediate-5: To an oven dried 250 mL round-bottom flask equipped with a magnetic stir bar taken Intermediate-4 (17.6 g, 20.86 mmol) and then suspended in 100 mL of DCM under argon atmosphere. 60 ml of 12 M HCl was added slowly and stirred for 3 h at room temperature. The reaction was monitored by TLC. After the reaction was completed, the liquid was separated, and the organic layer was washed with saturated sodium bicarbonate solution, dried with anhydrous magnesium sulfate, and solvents were removed in vacuo. The product was purified by SiO_2_ gel rapid column chromatography. Petroleum ether and ethyl acetate were used as eluents at 2:1 to obtain intermediate-5, which was colorless liquid. Yield: 53%, 7.50 g.

KEL1: To an oven dried 100 mL round bottom double opening flask equipped with a magnetic stir bar taken Intermediate-5 (2.0 g, 2.95 mmol) and then suspended in 40 mL of Anhydrous toluene under argon atmosphere. F (3-Dimethylaminopropane-1,2-diol, 1.0 g, 5.90 mmol) and Pyridinium p-Toluenesulfonate (PPTS, 1.68 g, 8.85 mmol) were added and used a Dean-Stark device with heating and refluxing at 135 ℃ for 20 h. The reaction was monitored by TLC. After the reaction was completed, it was cooled to room temperature. An equal volume of water was added to the reaction solution and then extracted with ethyl acetate (50 ml × 3). Solvents were removed in vacuo. The product was purified by SiO_2_ gel rapid column chromatography. DCM and methyl alcohol were used as eluents at 20:1 to obtain KEL1, which was colorless oily liquid. Yield: 4%, 94 mg. ^1^H NMR (600 MHz, Chloroform-*d*) δ 4.85 – 4.81 (m, 1H), 4.20 – 4.15 (m, 1H), 4.06 – 4.01 (m, 3H), 3.49 – 3.46 (m, 1H), 2.48 – 2.44 (m, 1H), 2.35f – 2.32 (m, 1H), 2.27 – 2.22 (m, 10H), 1.60 – 1.54 (m, 8H), 1.49 – 1.45 (m, 4H), 1.33 – 1.20 (m, 54H), 0.86 – 0.83 (m, 9H); ^13^C NMR (151 MHz, CDCl_3_) δ 173.90, 173.69, 112.58, 74.40, 74.36, 74.13, 69.06, 64.48, 62.62, 62.59, 46.22, 37.80, 37.55, 37.51, 37.30, 34.79, 34.40, 34.38, 34.24, 31.99, 31.94, 29.88, 29.83, 29.68, 29.66, 29.61, 29.58, 29.52, 29.47, 29.41, 29.38, 29.33, 29.31, 29.27, 29.25, 28.74, 26.01, 25.39, 25.24, 25.06, 25.06, 24.03, 23.70, 23.35, 22.74, 14.17; MS-ESI (m/z): 780.7 (M+H)^+^.


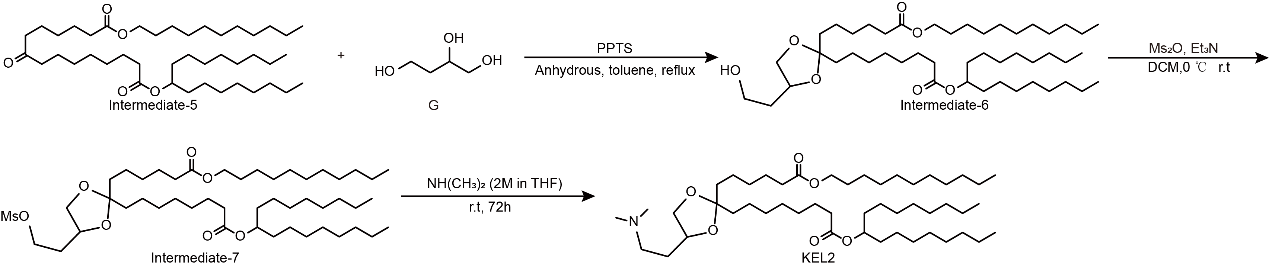


**Scheme S2.** Synthesis of KEL2.

**Synthesis of KEL2:** Intermediate-5: The synthesis of Intermediate-5 refers to the synthesis route of KEL1.

Intermediate-6: To an oven dried 100 mL round bottom double opening flask equipped with a magnetic stir bar taken Intermediate-5 (1.2 g, 1.84 mmol) and then suspended in 40 mL of Anhydrous toluene under argon atmosphere. G (1,2,4-Butanetriol, 0.39 g, 3.68 mmol) and PPTS (1.39 g, 5.53 mmol) were added and used a Dean-Stark device with heating and refluxing at 135 ℃ for 20 h. The reaction was monitored by TLC. After the reaction was completed, it was cooled to room temperature. An equal volume of water was added to the reaction solution and then extracted with ethyl acetate (50 ml × 3). Solvents were removed in vacuo. The product was purified by SiO_2_ gel rapid column chromatography. Petroleum ether and ethyl acetate were used as eluents at 10:1 to obtain Intermediate-6, which was colorless oily liquid. Yield: 53%, 0.75 g.

Intermediate-7: To an oven dried 25 mL round-bottom flask equipped with a magnetic stir bar taken Intermediate-6 (0.75 g, 0.98 mmol) and then suspended in 5 mL of an ultra-dry DCM under argon atmosphere at 0 ℃. Later, Methanesulfonic anhydride (0.36 g, 2.08 mmol) and Et_3_N (0.32 g, 3.12 mmol) were added and allowed to stir at room temperature for overnight. The reaction was monitored by TLC. After the reaction was completed, it was cooled to room temperature. An equal volume of water was added to the reaction solution to dilute, separate, and the organic layer was dried with anhydrous magnesium sulfate, filtered, the filtrate concentrated. The product was purified by SiO_2_ gel rapid column chromatography. Petroleum ether and ethyl acetate were used as eluents at 5:1 to obtain Intermediate-7, which was yellow oily liquid. Yield: 91%, 0.8 g.

KEL2: To an oven dried 50 mL round bottom double opening flask equipped with a magnetic stir bar taken Intermediate-7 (0.8 g, 0.95 mmol) under argon atmosphere. 20 ml of dimethylamine 2.0 M in THF solution and 0.5 ml Et_3_N were added and allowed to stir at room temperature for 72 h. The reaction was monitored by TLC. After the reaction was completed, an equal volume of water was added to the reaction solution and then extracted with ethyl acetate (50 ml × 3). Solvents were removed in vacuo. The product was purified by SiO_2_ gel rapid column chromatography. DCM and methyl alcohol were used as eluents at 40:1 to obtain KEL2, which was colorless oily liquid. Yield: 75%, 567 mg. ^1^H NMR (600 MHz, Chloroform-*d*) δ 4.82 – 4.78 (m, 1H), 4.04 – 3.96 (m, 4H), 3.42 – 3.39 (m, 1H), 2.34 (m, 1H), 2.30 – 2.24 (m, 1H), 2.24 – 2.16 (m, 10H), 1.78 – 1.70 (m, 1H), 1.67 – 1.59 (m, 1H), 1.58 – 1.39 (m, 14H), 1.34 – 1.07 (m, 53H), 0.82 – 0.80 (m, 6H); ^13^C NMR (151 MHz, CDCl_3_) δ 173.62, 173.41, 111.82, 74.63, 74.61, 73.88, 69.89, 64.25, 56.18, 45.28, 37.73, 37.48, 37.40, 37.20, 34.59, 34.19, 34.18, 34.09, 31.83, 31.78, 31.60, 29.70, 29.68, 29.52, 29.50, 29.45, 29.34, 29.32, 29.25, 29.20, 29.15, 29.09, 29.07, 28.59, 25.86, 25.23, 25.07, 24.89, 23.88, 23.58, 23.56, 23.24, 22.60, 22.58, 14.01; MS-ESI (m/z): 794.7 (M+H)^+^.


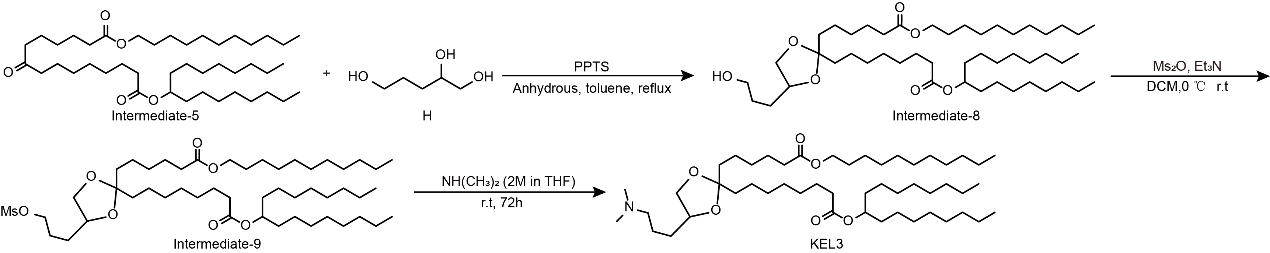


**Scheme S3.** Synthesis of KEL3.

**Synthesis of KEL3:** Intermediate-5: The synthesis of Intermediate-5 refers to the synthesis route of KEL1.

Intermediate-8: To an oven dried 100 mL round bottom double opening flask equipped with a magnetic stir bar taken Intermediate-5 (2.8 g, 4.16 mmol) and then suspended in 40 mL of Anhydrous toluene under argon atmosphere. H (1,2,5-Pentanetriol, 1.0 g, 8.32 mmol) and PPTS (1.0 g, 4.16 mmol) were added and used a Dean-Stark device with heating and refluxing at 135 ℃ for 20 h. The reaction was monitored by TLC. After the reaction was completed, it was cooled to room temperature. An equal volume of water was added to the reaction solution and then extracted with ethyl acetate (50 ml × 3). Solvents were removed in vacuo. The product was purified by SiO_2_ gel rapid column chromatography. Petroleum ether and ethyl acetate were used as eluents at 10:1 to obtain Intermediate-8, which was colorless oily liquid. Yield: 47%, 1.5 g.

KEL3: To an oven dried 25 mL round-bottom flask equipped with a magnetic stir bar taken Intermediate-8 (1.5 g, 1.93 mmol) and then suspended in 10 mL of an ultra-dry DCM under argon atmosphere at 0 ℃. Later, Methanesulfonic anhydride (0.68 g, 3.86 mmol) and Et_3_N (0.59 g, 5.79 mmol) were added and allowed to stir at room temperature for overnight. The reaction was monitored by TLC. After the reaction was completed, it was cooled to room temperature. Solvents were removed in vacuo. Crude Intermediate-9 was obtained. To an oven dried 50 mL round bottom double opening flask equipped with a magnetic stir bar taken crude Intermediate-9 under argon atmosphere. 20 ml of dimethylamine 2.0 M in THF solution and 0.5 ml Et_3_N were added and allowed to stir at room temperature for 72 h. The reaction was monitored by TLC. After the reaction was completed, an equal volume of water was added to the reaction solution and then extracted with ethyl acetate (50 ml × 3). Solvents were removed in vacuo. The product was purified by SiO_2_ gel rapid column chromatography. DCM and methyl alcohol were used as eluents at 40:1 to obtain KEL3, which was colorless oily liquid. Yield: 51%, 794 mg. ^1^H NMR (600 MHz, Chloroform-*d*) δ 4.80 – 4.76 (m, 1H), 3.99 – 3.92 (m, 4H), 3.37 – 3.34 (m, 1H), 2.24 – 2.15 (m, 6H), 2.15 (s, 6H), 1.58 – 1.38 (m, 20H), 1.29 – 1.15 (m, 50H), 0.81-0.78 (m, 7H); ^13^C NMR (151 MHz, CDCl_3_) δ 173.69, 173.47, 111.86, 76.08, 76.05, 73.92, 69.93, 69.91, 64.29, 59.48, 53.38, 45.24, 37.80, 37.56, 37.47, 37.25, 34.64, 34.24, 34.22, 34.13, 31.88, 31.83, 31.20, 29.76, 29.57, 29.55, 29.49, 29.47, 29.39, 29.37, 29.30, 29.27, 29.22, 29.20, 29.14, 29.11, 28.63, 25.90, 25.28, 25.12, 24.95, 23.95, 23.89, 23.64, 23.62, 23.29, 22.64, 22.62, 14.05; MS-ESI (m/z): 808.7 (M+H)^+^.

**
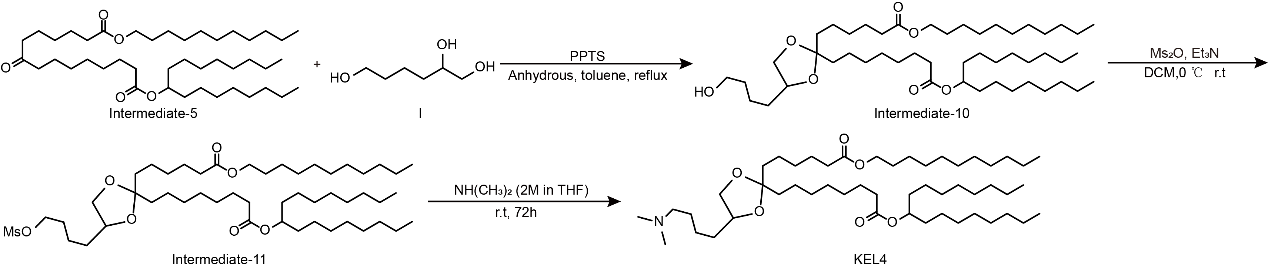
**

**Scheme S4.** Synthesis of KEL4.

**Synthesis of KEL4:** Intermediate-5: The synthesis of Intermediate-5 refers to the synthesis route of KEL1.

Intermediate-10: To an oven dried 100 mL round bottom double opening flask equipped with a magnetic stir bar taken Intermediate-5 (5.0 g, 7.36 mmol) and then suspended in 40 mL of Anhydrous toluene under argon atmosphere. I (1,2,6-Hexanetriol, 2.9 g, 22.1 mmol) and PPTS (1.9 g, 7.36 mmol) were added and used a Dean-Stark device with heating and refluxing at 135 ℃ for 20 h. The reaction was monitored by TLC. After the reaction was completed, it was cooled to room temperature. An equal volume of water was added to the reaction solution and then extracted with ethyl acetate (50 ml × 3). Solvents were removed in vacuo. The product was purified by SiO_2_ gel rapid column chromatography. Petroleum ether and ethyl acetate were used as eluents at 10:1 to obtain Intermediate-11, which was yellow oily liquid. Yield: 35%, 2.0 g.

KEL4: To an oven dried 25 mL round-bottom flask equipped with a magnetic stir bar taken Intermediate-10 (0.5 g, 0.63 mmol) and then suspended in 10 mL of an ultra-dry DCM under argon atmosphere at 0 ℃. Later, Methanesulfonic anhydride (0.22 g, 1.26 mmol) and Et_3_N (0.19 g, 1.89 mmol) were added and allowed to stir at room temperature for overnight. The reaction was monitored by TLC. After the reaction was completed, it was cooled to room temperature. Solvents were removed in vacuo. Crude Intermediate-11 was obtained. To an oven dried 50 mL round bottom double opening flask equipped with a magnetic stir bar taken crude Intermediate-11 under argon atmosphere. 20 ml of dimethylamine 2.0 M in THF solution and 0.5 ml Et_3_N were added and allowed to stir at room temperature for 72 h. The reaction was monitored by TLC. After the reaction was complete. An equal volume of water was added to the reaction solution and then extracted with ethyl acetate (50 ml × 3). Solvents were removed in vacuo. The product was purified by SiO_2_ gel rapid column chromatography. DCM and methyl alcohol were used as eluents at 40:1 to obtain KEL4, which was oily liquid. Yield: 43%, 222 mg. ^1^H NMR (600 MHz, Chloroform-*d*) δ 4.91 – 4.79 (m, 1H), 4.09 – 3.95 (m, 4H), 3.45 – 3.34 (m, 1H), 2.33 (t, *J* = 6.5 Hz, 2H), 2.30 – 2.22 (m, 10H), 1.66 – 1.55 (m, 9H), 1.53 – 1.41 (m, 8H), 1.38 – 1.15 (m, 55H), 0.87 (t, *J* = 7.0 Hz, 9H); ^13^C NMR (151 MHz, Chloroform-*d*) δ 173.90,173.66, 111.89, 76.20, 76.16, 74.09, 70.03, 64.42, 59.48, 45.20, 37.62, 37.56, 34.73, 34.73, 34.34, 34.32, 34.15, 33.31, 31.90, 31.85, 29.82, 29.79, 29.69, 29.58, 29.52, 29.49, 29.45, 29.43, 29.32, 29.25, 29.22, 29.18, 28.66, 27.37, 25.92, 25.31, 25.16, 24.99, 24.01, 23.71, 23.68, 23.33, 22.66, 14.09; MS-ESI (m/z): 822.9(M+H)^+^.


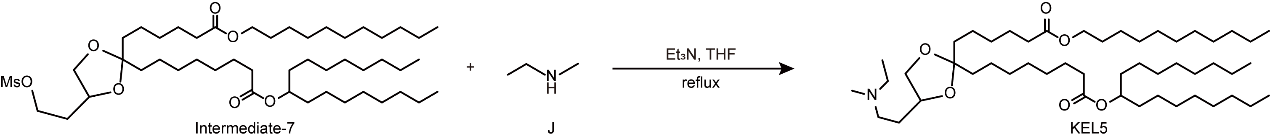


**Scheme S5.** Synthesis of KEL5.

**Synthesis of KEL5:** Intermediate-7: The synthesis of Intermediate-7 refers to the synthesis route of KEL2.

KEL5: To an oven dried 50 mL round-bottom flask equipped with a magnetic stir bar taken Intermediate-7 (0.3 g, 0.35 mmol) and then suspended in 15 mL of Ultra dry THF under argon atmosphere. J (Methyl ethylamine, 63 mg, 1.07 mmol) and Et_3_N (109 mg, 1.07 mmol) were added and refluxed at 60 ℃ for 18 h. The reaction was monitored by TLC. After the reaction was completed, it was cooled to room temperature. An equal volume of water was added to the reaction solution and then extracted with ethyl acetate (15 ml × 3). Solvents were removed in vacuo. The product was purified by SiO_2_ gel rapid column chromatography. DCM and methyl alcohol were used as eluents at 40:1 to obtain KEL5, which was oily liquid. Yield: 51%, 144 mg. ^1^H NMR (600 MHz, CDCl_3_) δ 4.81 – 4.77 (m, 1H), 4.03-3.96 (m, 4H), 3.39 (t, *J* = 7.7 Hz, 1H), 2.45 – 2.31 (m, 4H), 2.23 – 2.15 (m, 7H), 1.77 – 1.71 (m, 1H), 1.65 – 1.59 (m, 1H), 1.56 – 1.52 (m, 6H), 1.43 (d, *J* = 7.2 Hz, 4H), 1.28 – 1.18 (m, 56H), 0.99 (t, *J* = 7.1 Hz, 3H), 0.80 (t, *J* = 7.0 Hz, 9H); ^13^C NMR (151 MHz, CDCl_3_) δ 173.72, 173.51, 111.83, 74.85, 74.81, 73.96, 69.99, 64.33, 53.70, 51.43, 41.49, 37.80, 37.56, 37.48, 37.28, 34.66, 34.26, 34.25, 34.15, 31.90, 31.85, 31.30, 29.77, 29.59, 29.57, 29.51, 29.49, 29.41, 29.38, 29.32, 29.29, 29.24, 29.22, 29.16, 29.13, 28.65, 25.92, 25.30, 25.13, 24.97, 23.96, 23.66, 23.31, 22.65, 14.08, 12.09; MS-ESI (m/z): 808.7(M+H)^+^.


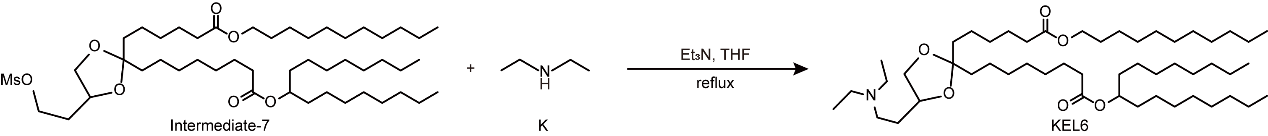


**Scheme S6.** Synthesis of KEL6.

**Synthesis of KEL6:** Intermediate-7: The synthesis of Intermediate-7 refers to the synthesis route of KEL2.

KEL6: To an oven dried 50 mL round-bottom flask equipped with a magnetic stir bar taken Intermediate-7 (200 mg, 0.24 mmol) and then suspended in 15 mL of Ultra dry THF under argon atmosphere. K (Diethylamine, 52 mg, 0.71 mmol) and Et_3_N (72 mg, 0.71 mmol) were added and refluxed at 60 ℃ for 18 h. The reaction was monitored by TLC. After the reaction was completed, it was cooled to room temperature. An equal volume of water was added to the reaction solution and then extracted with ethyl acetate (15 ml × 3). Solvents were removed in vacuo. The product was purified by SiO_2_ gel rapid column chromatography. DCM and methyl alcohol were used as eluents at 40:1 to obtain KEL5, which was oily liquid. Yield: 27%, 53 mg. ^1^H NMR (600 MHz, CDCl_3_) δ 4.86 – 4.81 (m, 1H), 4.08 – 4.01 (m, 4H), 3.49 – 3.43 (m, 1H), 2.75 – 2.65 (m, 6H), 2.25 (dt, *J* = 10.5, 7.5 Hz, 4H), 1.85 – 1.76 (m, 2H), 1.62 – 1.57 (m, 6H), 1.50 – 1.44 (m, 4H), 1.37 – 1.23 (m, 56H), 1.12 (t, *J* = 7.2 Hz, 6H), 0.85 (t, *J* = 7.1, 2.4 Hz, 9H); ^13^C NMR (151 MHz, CDCl_3_) δ 173.95, 173.73, 112.22, 74.59, 74.55, 74.17, 69.94, 64.52, 49.36, 46.95, 37.84, 37.59, 37.42, 37.21, 34.80, 34.39, 34.24, 31.99, 31.95, 30.30, 29.87, 29.69, 29.67, 29.62, 29.59, 29.50, 29.47, 29.42, 29.35, 29.32, 29.29, 29.26, 28.74, 26.02, 25.40, 25.24, 25.07, 24.09, 23.82, 23.76, 23.47, 22.75, 14.20, 10.74; MS-ESI (m/z): 822.7(M+H)^+^.


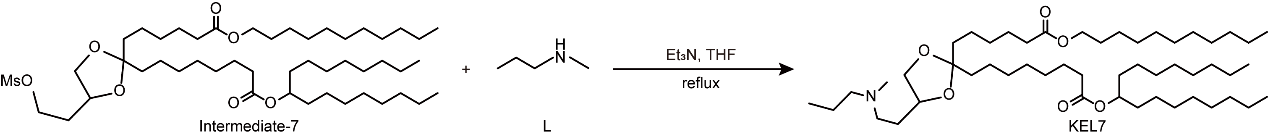


**Scheme S7.** Synthesis of KEL7.

**Synthesis of KEL7:** Intermediate-7: The synthesis of Intermediate-7 refers to the synthesis route of KEL2.

KEL7: To an oven dried 50 mL round-bottom flask equipped with a magnetic stir bar taken Intermediate-7 (500 mg, 0.92 mmol) and then suspended in 15 mL of Ultra dry THF under argon atmosphere. L (N-Methylpropylamine, 201 mg, 2.75 mmol) and Et_3_N (278 mg, 2.75 mmol) were added and refluxed at 60 ℃ for 18 h. The reaction was monitored by TLC. After the reaction was completed, it was cooled to room temperature. An equal volume of water was added to the reaction solution and then extracted with ethyl acetate (15 ml × 3). Solvents were removed in vacuo. The product was purified by SiO_2_ gel rapid column chromatography. DCM and methyl alcohol were used as eluents at 40:1 to obtain KEL5, which was oily liquid. Yield: 17%, 132 mg. ^1^H NMR (600 MHz, CDCl_3_) δ 4.82 – 4.80 (m, 1H), 4.05 – 3.99 (m, 4H), 3.42 (t, *J* = 7.7 Hz, 1H), 2.51 – 2.40 (m, 2H), 2.33 (t, *J* = 7.6 Hz, 2H), 2.25 – 2.21 (m, 7H), 1.80 – 1.66 (m, 2H), 1.58 – 1.55 (m, 6H), 1.50 – 1.45 (m, 5H), 1.34 – 1.21 (m, 55H), 0.87 – 0.81 (m, 12H); ^13^C NMR (151 MHz, CDCl_3_) δ 173.86, 173.64, 111.97, 74.82, 74.78, 74.09, 70.02, 64.45, 59.66, 54.18, 41.99, 37.87, 37.62, 37.52, 37.32, 34.76, 34.35, 34.22, 31.97, 31.92, 31.06, 29.85, 29.83, 29.64, 29.58, 29.56, 29.49, 29.46, 29.39, 29.31, 29.29, 29.25, 29.22, 28.72, 25.99, 25.37, 25.21, 25.04, 24.05, 23.75, 23.72, 23.41, 22.73, 22.71, 20.09, 14.15, 11.84; MS-ESI (m/z): 822.7 (M+H)^+^.


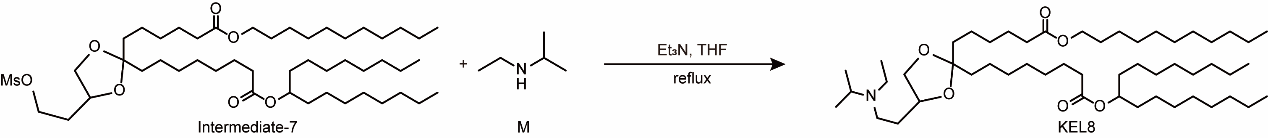


**Scheme S8.** Synthesis of KEL8.

**Synthesis of KEL8:** Intermediate-7: The synthesis of Intermediate-7 refers to the synthesis route of KEL2.

KEL8: To an oven dried 50 mL round-bottom flask equipped with a magnetic stir bar taken Intermediate-7 (300 mg, 0.36 mmol) and then suspended in 15 mL of Ultra dry THF under argon atmosphere. K (N-Ethylisopropylamine, 92 mg, 1.06 mmol) and Et_3_N (108 mg, 1.06 mmol) were added and refluxed at 60 ℃ for 18 h. The reaction was monitored by TLC. After the reaction was completed, it was cooled to room temperature. An equal volume of water was added to the reaction solution and then extracted with ethyl acetate (15 ml × 3). Solvents were removed in vacuo. The product was purified by SiO_2_ gel rapid column chromatography. DCM and methyl alcohol were used as eluents at 40:1 to obtain KEL5, which was oily liquid. Yield: 33%, 100 mg. ^1^H NMR (600 MHz, CDCl_3_) δ 4.86 – 4.82 (m, 1H), 4.11 – 4.02 (m, 4H), 3.47 (t, *J* = 7.6 Hz, 1H), 3.18 – 2.68 (m, 5H), 2.26 (dt, *J* = 10.4, 7.5 Hz, 4H), 1.63 – 1.44 (m, 14H), 1.39 – 1.09 (m, 63H), 0.87 – 0.84 (m, 9H); ^13^C NMR (151 MHz, CDCl_3_) δ 173.95, 173.73, 112.18, 74.58, 74.19, 69.97, 64.54, 46.37, 44.49, 37.86, 37.61, 37.44, 37.23, 34.82, 34.41, 34.25, 32.01, 31.97, 29.90, 29.87, 29.80, 29.70, 29.68, 29.63, 29.60, 29.50, 29.43, 29.36, 29.33, 29.28, 28.76, 26.03, 25.42, 25.25, 25.10, 25.08, 24.12, 23.85, 23.79, 23.50, 22.78, 22.76, 18.33, 17.19, 14.20, 0.09; MS-ESI (m/z): 836.7(M+H)^+^.


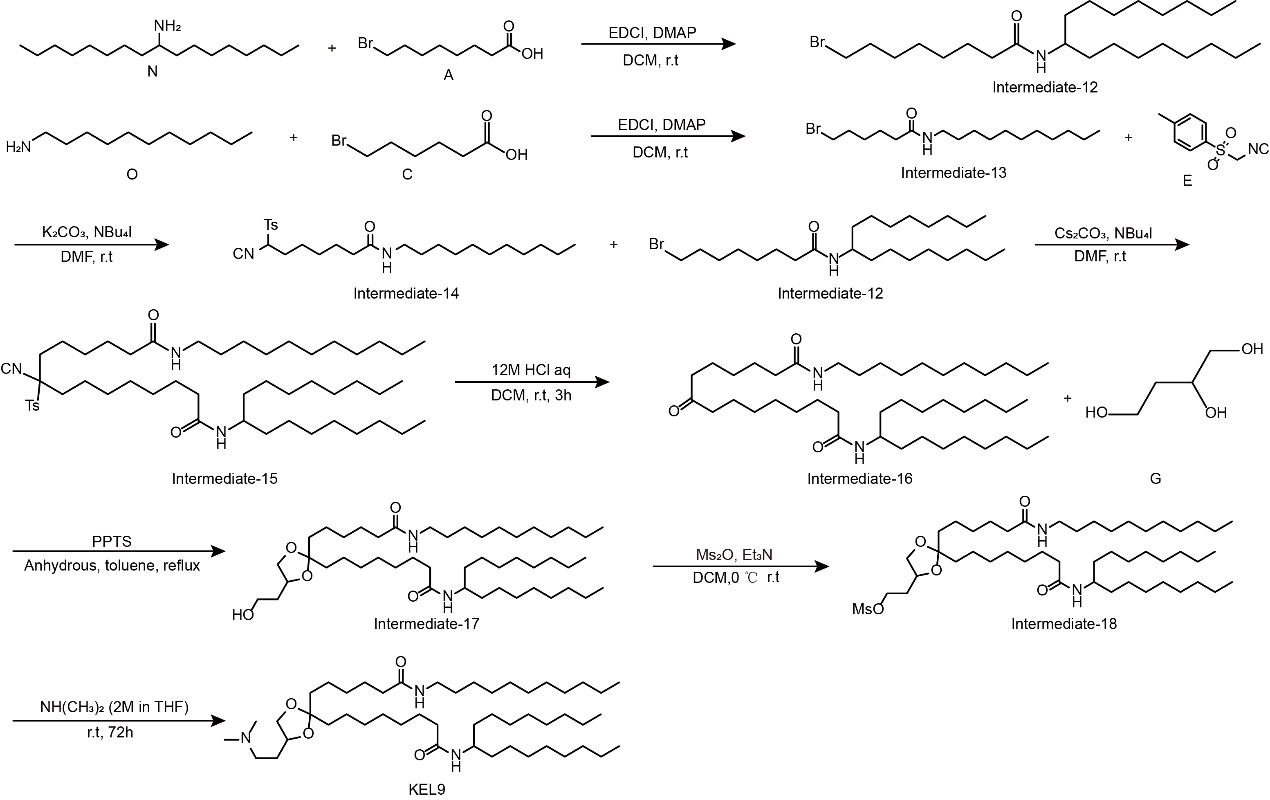


**Scheme S9.** Synthesis of KEL9.

**Synthesis of KEL9:** Intermediate-12: To an oven dried 250 mL round-bottom flask equipped with a magnetic stir bar taken A (8-Bromooctanoic acid, 5.24 g, 23.5 mmol) and then suspended in 100 mL of DCM under argon atmosphere. EDCI (4.9 g, 25.4 mmol) and DMAP (0.96 g, 7.8 mmol) were added and allowed to stir for 15 min. N (Heptadecan-9-amine, 5.0 g, 19.6 mmol) was then added and allowed to stir at room temperature for overnight. After the reaction was completed, an equal volume of saturated sodium bicarbonate solution was added to the reaction solution to dilute and separate, and anhydrous magnesium sulfate was added to the organic layer to dry, filter, and solvents were removed in vacuo. The product was purified by SiO_2_ gel rapid column chromatography. DCM and methyl alcohol were used as eluents at 20:1 to obtain intermediate 12, which was white solid. Yield: 90%, 8.12 g.

Intermediate-13: To an oven dried 250 mL round-bottom flask equipped with a magnetic stir bar taken C (6-Bromohexanoic acid, 9.6 g, 49 mmol) and then suspended in 10 mL of DCM under argon atmosphere. EDCI (10.2 g, 53.1 mmol) and DMAP (2.0 g, 16.4 mmol) were then added and allowed to stir for 15 min. O (1-Aminoundecane, 7.0 g, 40.8 mmol) was added and allowed to stir at room temperature for overnight. After the reaction was completed, an equal volume of saturated sodium bicarbonate solution was added to the reaction solution to dilute and separate, and anhydrous magnesium sulfate was added to the organic layer to dry, filter, and solvents were removed in vacuo. The product was purified by SiO_2_ gel rapid column chromatography. Petroleum ether and ethyl acetate were used as eluents at 30:1 to obtain intermediate 2, which was white solid. Yield: 86%, 12.20 g.

Intermediate-14: To an oven dried 250 mL round-bottom flask equipped with a magnetic stir bar taken Intermediate-13 (12.2 g, 35.1 mmol) and then suspended in 100 mL of DMF under argon atmosphere. E (13.7 g, 70.28 mmol), K_2_CO_3_(14.5 g, 105.4 mmol) and NBu_4_I (13.0 g, 35.1 mmol) were then added and allowed to stir at room temperature for 12 h. The reaction was monitored by TLC. After the reaction was completed, an equal volume of water was added to the reaction solution, diluted, extracted with ethyl acetate (50 ml × 3), and solvents were removed in vacuo. The product was purified by SiO_2_ gel rapid column chromatography. Petroleum ether and ethyl acetate were used as eluents at 2:1 to obtain intermediate 14, which was white solid. Yield: 62%, 9.9 g.

Intermediate-15: To an oven dried 250 mL round-bottom flask equipped with a magnetic stir bar taken Intermediate-14 (9.9 g, 21.4 mmol) and then suspended in 100 mL of DMF under argon atmosphere. Intermediate-12 (11.1 g, 32.1 mmol), Cs_2_CO_3_ (14.0 g, 86.6 mmol) and NBu_4_I (8.0 g, 32.1 mmol) were then added and allowed to stir at room temperature for 12 h. The reaction was monitored by TLC. After the reaction was completed, an equal volume of water was added to the reaction solution, diluted, extracted with ethyl acetate (50 ml × 3), and solvents were removed in vacuo. The product was purified by SiO_2_ gel rapid column chromatography. Petroleum ether and ethyl acetate were used as eluents at 2:1 to obtain intermediate 4, which was colorless oily liquid. Yield: 67%, 12.0 g.

Intermediate-16: To an oven dried 250 mL round-bottom flask equipped with a magnetic stir bar taken Intermediate-15 (12.0 g, 22.2 mmol) and then suspended in 100 mL of DCM under argon atmosphere. 60 ml of 12 M HCl was added slowly and stirred for 3 h at room temperature. The reaction was monitored by TLC. After the reaction was completed, the liquid was separated, and the organic layer was washed with saturated sodium bicarbonate solution, dried with anhydrous magnesium sulfate, and solvents were removed in vacuo. The product was purified by SiO_2_ gel rapid column chromatography. Petroleum ether and ethyl acetate were used as eluents at 2:1 to obtain intermediate-5, which was white solid. Yield: 62%, 9.3 g.

Intermediate-17: To an oven dried 100 mL round bottom double opening flask equipped with a magnetic stir bar taken Intermediate-16 (3.0 g, 4.4 mmol) and then suspended in 40 mL of Anhydrous toluene under argon atmosphere. G (1,2,4-Butanetriol, 1.42 g, 13.3 mmol) and PPTS (1.34 g, 6.65 mmol) were added and used a Dean-Stark device with heating and refluxing at 135 °C for 20 h. The reaction was monitored by TLC. After the reaction was completed, it was cooled to room temperature. An equal volume of water was added to the reaction solution and then extracted with ethyl acetate (50 ml × 3). Solvents were removed in vacuo. The product was purified by SiO_2_ gel rapid column chromatography. Petroleum ether and ethyl acetate were used as eluents at 10:1 to obtain Intermediate-6, which was colorless oily liquid. Yield: 38%, 1.3 g.

KEL9: To an oven dried 25 mL round-bottom flask equipped with a magnetic stir bar taken Intermediate-17 (1.3 g, 1.7 mmol) and then suspended in 10 mL of an ultra-dry DCM under argon atmosphere at 0 ℃. Later, Methanesulfonic anhydride (0.59 g, 3.4 mmol) and Et_3_N (0.51 g, 5.2 mmol) were added and allowed to stir at room temperature for overnight. The reaction was monitored by TLC. After the reaction was completed, it was cooled to room temperature. Solvents were removed in vacuo. Crude Intermediate-18 was obtained. To an oven dried 50 mL round bottom double opening flask equipped with a magnetic stir bar taken crude Intermediate-18 under argon atmosphere. 20 ml of dimethylamine 2.0 M in THF solution and 0.5 ml Et_3_N were added and allowed to stir at room temperature for 72 h. The reaction was monitored by TLC. After the reaction was completed, an equal volume of water was added to the reaction solution and then extracted with ethyl acetate (50 ml × 3). Solvents were removed in vacuo. The product was purified by SiO_2_ gel rapid column chromatography. DCM, methyl alcohol and ammonia water were used as eluents at 300:10:0.5 to obtain KEL9, which was yellow oily liquid. Yield: 22%, 300 mg. ^1^H NMR (500 MHz, CDCl_3_) δ 5.83 – 5.70 (m, 1H), 5.34 (t, *J* = 9.1 Hz, 1H), 4.05 – 3.97 (m, 2H), 3.87 – 3.80 (m, 1H), 3.41 (t, *J* = 7.4 Hz, 1H), 3.16 (q, *J* = 6.7 Hz, 2H), 2.42 – 2.34 (m, 1H), 2.28 – 2.23 (m, 1H), 2.19 (d, *J* = 3.3 Hz, 6H), 2.09 (t, *J* = 7.6 Hz, 4H), 1.78 – 1.70 (m, 1H), 1.66 – 1.47 (m, 8H), 1.44 – 1.39 (m, 3H), 1.30 – 1.19 (m, 56H), 0.82 (t, *J* = 6.9, 2.4 Hz, 9H); ^13^C NMR (126 MHz, CDCl_3_) δ 172.97, 172.62, 111.98, 111.94, 74.72, 74.68, 69.97, 56.31, 49.09, 45.48, 45.45, 39.53, 37.71, 37.49, 37.38, 37.25, 37.11, 36.74, 36.72, 35.33, 31.93, 31.90, 31.75, 29.73, 29.63, 29.59, 29.48, 29.36, 29.30, 29.24, 26.99, 25.99, 25.94, 25.80, 25.76, 23.92, 23.77, 23.60, 23.40, 22.69, 14.14; MS-ESI (m/z):792.7 (M+H)^+^.


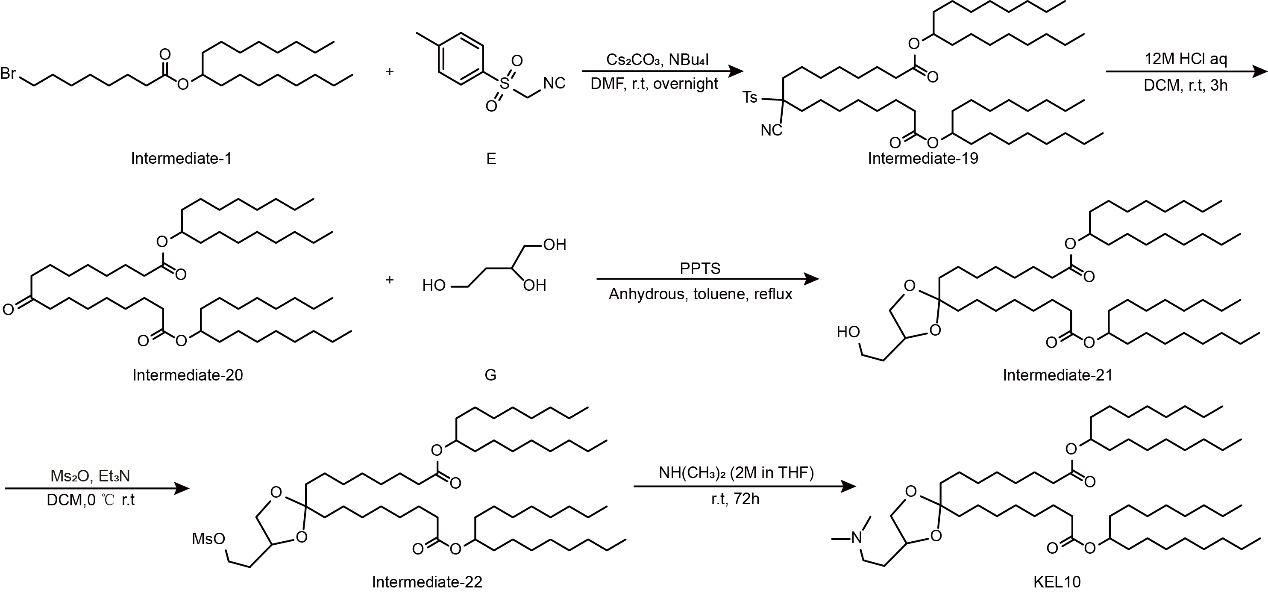


**Scheme S10.** Synthesis of KEL10.

**Synthesis of KEL10:** Intermediate-19: To an oven dried 250 mL round-bottom flask equipped with a magnetic stir bar taken Intermediate-1 (15.0 g, 32.6 mmol) and then suspended in 100 mL of DMF under argon atmosphere. E (2.2 g, 10.9 mmol), Cs_2_CO_3_ (10.6 g, 32.6 mmol) and NBu_4_I (4.0 g, 10.9 mmol) were added and allowed to stir at room temperature for 12 h. The reaction was monitored by TLC. After the reaction was completed, an equal volume of water was added to the reaction solution, diluted, extracted with ethyl acetate (50 ml × 3), and solvents were removed in vacuo. The product was purified by SiO_2_ gel rapid column chromatography. Petroleum ether and ethyl acetate were used as eluents at 30:1 to obtain intermediate 19, which was colorless oily liquid. Yield: 76%, 7.9 g.

Intermediate-20: To an oven dried 250 mL round-bottom flask equipped with a magnetic stir bar taken Intermediate-19 (7.9 g, 8.3 mmol) and then suspended in 100 mL of DCM under argon atmosphere. 60 ml of 12 M HCl was added slowly and stirred for 3 h at room temperature. The reaction was monitored by TLC. After the reaction was completed, the liquid was separated, and the organic layer was washed with saturated sodium bicarbonate solution, dried with anhydrous magnesium sulfate, and solvents were removed in vacuo. The product was purified by SiO_2_ gel rapid column chromatography. Petroleum ether and ethyl acetate were used as eluents at 2:1 to obtain intermediate-20, which was colorless liquid. Yield: 90%, 5.9 g.

Intermediate-21: To an oven dried 100 mL round bottom double opening flask equipped with a magnetic stir bar taken Intermediate-20 (5.9 g, 7.4 mmol) and then suspended in 40 mL of Anhydrous toluene under argon atmosphere. G (2.4 g, 22.35 mmol) and PPTS (2.9 g, 11.7 mmol) were added and used a Dean-Stark device with heating and refluxing at 135 ℃ for 20 h. The reaction was monitored by TLC. After the reaction was completed, it was cooled to room temperature. An equal volume of water was added to the reaction solution and then extracted with ethyl acetate (50 ml × 3). Solvents were removed in vacuo. The product was purified by SiO_2_ gel rapid column chromatography. Petroleum ether and ethyl acetate were used as eluents at 10:1 to obtain Intermediate-21, which was colorless oily liquid. Yield: 45%, 2.9 g.

KEL10: To an oven dried 25 mL round-bottom flask equipped with a magnetic stir bar taken Intermediate-21 (2.9 g, 3.4 mmol) and then suspended in 10 mL of an ultra-dry DCM under argon atmosphere at 0 ℃. Later, Methanesulfonic anhydride (1.2 g, 6.8 mmol) and Et_3_N (1.1 g, 10.2 mmol) were added and allowed to stir at room temperature for overnight. The reaction was monitored by TLC. After the reaction was completed, it was cooled to room temperature. Solvents were removed in vacuo, crude Intermediate-22 were obtained. To an oven dried 50 mL round bottom double opening flask equipped with a magnetic stir bar taken crude Intermediate-22 under argon atmosphere. 20 ml of dimethylamine 2.0 M in THF solution and 0.5 ml Et_3_N were added and allowed to stir at room temperature for 72 h. The reaction was monitored by TLC. After the reaction was completed, an equal volume of water was added to the reaction solution and then extracted with ethyl acetate (50 ml × 3). Solvents were removed in vacuo. The product was purified by SiO_2_ gel rapid column chromatography. DCM and methyl alcohol were used as eluents at 40:1 to obtain KEL10, which was oily liquid. Yield: 15%, 439 mg. ^1^H NMR (500 MHz, CDCl_3_) δ 4.84 – 4.80 (m, 2H), 4.08 – 4.00 (m, 2H), 3.44 (t, *J* = 7.4 Hz, 1H), 2.45 – 2.37 (m, 1H), 2.34 – 2.28 (m, 1H), 2.22 (d, *J* = 6.7 Hz, 10H), 1.82 – 1.75 (m, 1H), 1.70 – 1.63 (m, 1H), 1.57 – 1.52 (m, 3H), 1.46 (d, *J* = 6.1 Hz, 8H), 1.32 – 1.22 (m, 69H), 0.84 (t, *J* = 6.9 Hz, 12H); ^13^C NMR (126 MHz, CDCl_3_) δ 173.69, 112.11, 74.73, 74.10, 70.02, 56.33, 45.42, 37.85, 37.53, 34.78, 34.23, 31.94, 31.70, 29.87, 29.60, 29.31, 29.27, 29.24, 25.39, 25.23, 24.05, 23.75, 22.74, 14.18; MS-ESI (m/z): 906.8 (M+H)^+^.


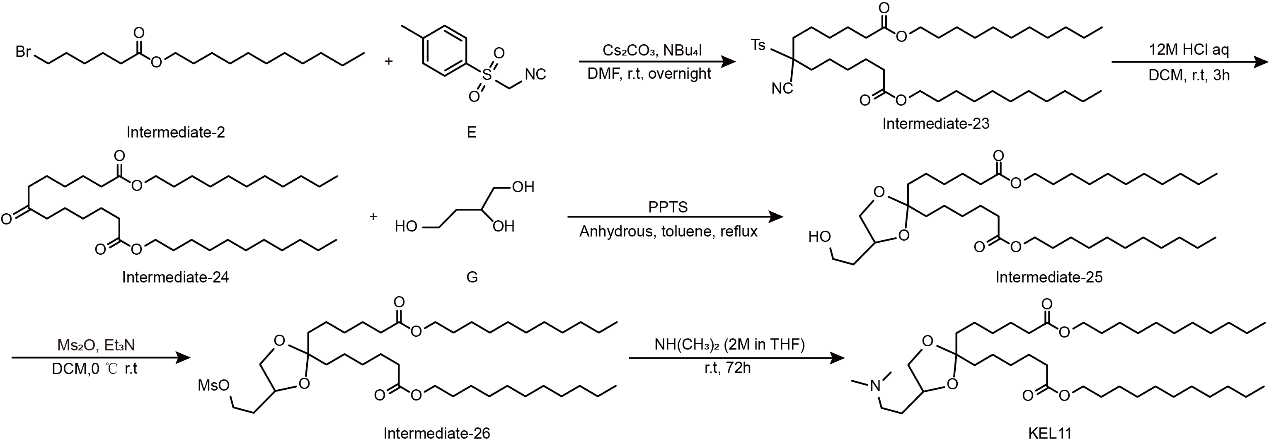


**Scheme S11.** Synthesis of KEL11.

**Synthesis of KEL11:** Intermediate-23: To an oven dried 250 mL round-bottom flask equipped with a magnetic stir bar taken Intermediate-2 (17.0 g, 55.3 mmol) and then suspended in 100 mL of DMF under argon atmosphere. E (3.6 g, 18.4 mmol), Cs_2_CO_3_ (36.0 g, 110.6 mmol) and NBu_4_I (20.0 g, 55.3 mmol) were added and allowed to stir at room temperature for 12 h. The reaction was monitored by TLC. After the reaction was completed, an equal volume of water was added to the reaction solution, diluted, extracted with ethyl acetate (50 ml × 3), and solvents were removed in vacuo. The product was purified by SiO_2_ gel rapid column chromatography. Petroleum ether and ethyl acetate were used as eluents at 30:1 to obtain intermediate 23, which was white solid. Yield: 41%, 5.45 g.

Intermediate-24: To an oven dried 250 mL round-bottom flask equipped with a magnetic stir bar taken Intermediate-23 (5.45 g, 7.5 mmol) and then suspended in 100 mL of DCM under argon atmosphere. 60 ml of 12 M HCl was added slowly and stirred for 3 h at room temperature. The reaction was monitored by TLC. After the reaction was completed, the liquid was separated, and the organic layer was washed with saturated sodium bicarbonate solution, dried with anhydrous magnesium sulfate, and solvents were removed in vacuo. The product was purified by SiO_2_ gel rapid column chromatography. Petroleum ether and ethyl acetate were used as eluents at 2:1 to obtain intermediate-24, which was colorless liquid. Yield: 48%, 2.0 g.

Intermediate-25: To an oven dried 100 mL round bottom double opening flask equipped with a magnetic stir bar taken Intermediate-24 (2.0 g, 3.5 mmol) and then suspended in 40 mL of anhydrous toluene under argon atmosphere. G (1.1 g, 10.6 mmol) and PPTS (1.3 g, 5.3 mmol) were added and used a Dean-Stark device with heating and refluxing at 135 ℃for 20 h. The reaction was monitored by TLC. After the reaction was completed, it was cooled to room temperature. An equal volume of water was added to the reaction solution and then extracted with ethyl acetate (50 ml × 3). Solvents were removed in vacuo. The product was purified by SiO_2_ gel rapid column chromatography. Petroleum ether and ethyl acetate were used as eluents at 10:1 to obtain Intermediate-25, which was colorless oily liquid. Yield: 39%, 0.9 g.

KEL11: To an oven dried 25 mL round-bottom flask equipped with a magnetic stir bar taken Intermediate-25 (0.9 g, 1.4 mmol) and then suspended in 10 mL of an ultra-dry DCM under argon atmosphere at 0 ℃. Later, Methanesulfonic anhydride (0.49 g, 2.8 mmol) and Et_3_N (0.42 g, 4.2 mmol) were added and allowed to stir at room temperature for overnight. The reaction was monitored by TLC. After the reaction was completed, it was cooled to room temperature. Solvents were removed in vacuo. Crude Intermediate-26 was obtained. To an oven dried 50 mL round bottom double opening flask equipped with a magnetic stir bar taken crude Intermediate-26 under argon atmosphere. 20 ml of dimethylamine 2.0 M in THF solution and 0.5 ml Et_3_N was added and allowed to stir at room temperature for 72 h. The reaction was monitored by TLC. After the reaction was completed, an equal volume of water was added to the reaction solution and then extracted with ethyl acetate (50 ml × 3). Solvents were removed in vacuo. The product was purified by SiO_2_ gel rapid column chromatography. DCM and methyl alcohol were used as eluents at 40:1 to obtain KEL11, which was yellow oily liquid. Yield: 57%, 540 mg. ^1^H NMR (500 MHz, CDCl_3_) δ 4.07 – 4.01 (m, 6H), 3.44 (t, *J* = 7.4 Hz, 1H), 2.46 – 2.39 (m, 1H), 2.35 – 2.30 (m, 1H), 2.25 (d, *J* = 7.3 Hz, 10H), 1.82 – 1.75 (m, 1H), 1.70 – 1.65 (m, 1H), 1.61 – 1.53 (m, 11H), 1.29 – 1.23 (m, 41H), 0.85 (t, *J* = 6.9 Hz, 6H); ^13^C NMR (126 MHz, CDCl_3_) δ 173.94, 111.93, 74.78, 70.07, 64.50, 56.33, 45.44, 37.67, 37.39, 34.39, 31.99, 31.67, 29.68, 29.61, 29.50, 29.42, 29.34, 28.74, 26.01, 25.06, 23.75, 23.42, 22.77, 14.21; MS-ESI (m/z): 682.6 (M+H)^+^.


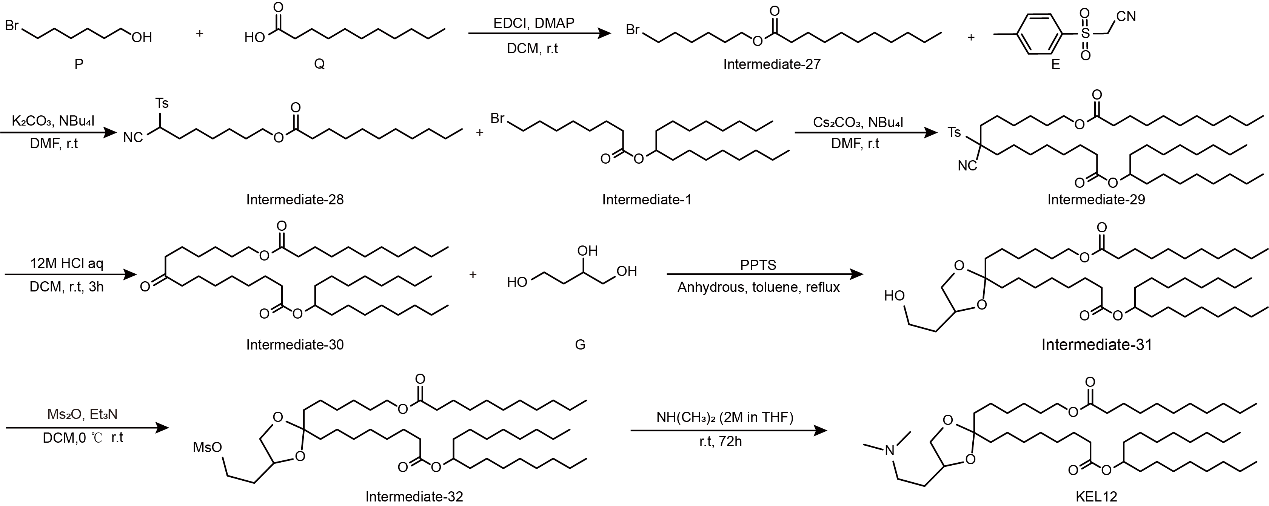


**Scheme S12.** Synthesis of KEL12.

**Synthesis of KEL12:** Intermediate-27: To an oven dried 500 mL round-bottom flask equipped with a magnetic stir bar taken Q (Hendecanoic acid 37.0 g, 0.20 mol) and then suspended in 200 mL of DCM under argon atmosphere. EDCI (41.0 g, 0.22 mol) and DMAP (8.2 g, 0.07 mol) were then added and allowed to stir for 15 min. P (6-Bromo-1-hexanol, 30.0 g, 0.17 mol) was then added and allowed to stir at room temperature for overnight. After the reaction was completed, an equal volume of saturated sodium bicarbonate solution was added to the reaction solution to dilute and separate, and anhydrous magnesium sulfate was added to the organic layer to dry, filter, and solvents were removed in vacuo. The product was purified by SiO_2_ gel rapid column chromatography. Petroleum ether and ethyl acetate were used as eluents at 30:1 to obtain intermediate-27, which was colorless oily liquid. Yield: 78%, 45.0 g.

Intermediate-28: To an oven dried 250 mL round-bottom flask equipped with a magnetic stir bar taken Intermediate-27 (10.0 g, 28.7 mmol) and then suspended in 100 mL of DMF under argon atmosphere. E (10.8 g, 56.0 mmol), K_2_CO_3_ (12.0 g, 84.0 mmol) and NBu_4_I (4.8 g, 14.0 mmol) were then added and allowed to stir at room temperature for 12 h. The reaction was monitored by TLC. After the reaction was completed, an equal volume of water was added to the reaction solution, diluted, extracted with ethyl acetate (50 ml × 3), and solvents were removed in vacuo. The product was purified by SiO_2_ gel rapid column chromatography. Petroleum ether and ethyl acetate were used as eluents at 15:1 to obtain intermediate-28, which was white solid. Yield: 70%, 9.1 g.

Intermediate-29: To an oven dried 250 mL round-bottom flask equipped with a magnetic stir bar taken Intermediate-28 (9.1 g, 19.6 mmol) and then suspended in 100 mL of DMF under argon atmosphere. Intermediate-1 (18 g, 39.2 mmol), Cs_2_CO_3_ (19.1 g, 58.8 mmol) and NBu_4_I (3.7 g, 9.8 mmol) were then added and allowed to stir at room temperature for 12 h. The reaction was monitored by TLC. After the reaction was completed, an equal volume of water was added to the reaction solution, diluted, extracted with ethyl acetate (50 ml × 3), and solvents were removed in vacuo. The product was purified by SiO_2_ gel rapid column chromatography. Petroleum ether and ethyl acetate were used as eluents at 3:1 to obtain intermediate 29, which was white solid. Yield: 50%, 8.3 g.

Intermediate-30: To an oven dried 250 mL round-bottom flask equipped with a magnetic stir bar taken Intermediate-29 (8.3 g, 9.8 mmol) and then suspended in 100 mL of DCM under argon atmosphere. 60 ml of 12 M HCl was added slowly and stirred for 3 h at room temperature. The reaction was monitored by TLC. After the reaction was completed, the liquid was separated, and the organic layer was washed with saturated sodium bicarbonate solution, dried with anhydrous magnesium sulfate, and solvents were removed in vacuo. The product was purified by SiO_2_ gel rapid column chromatography. Petroleum ether and ethyl acetate were used as eluents at 2:1 to obtain intermediate-30, which was colorless liquid. Yield: 50%, 3.3 g.

Intermediate-31: To an oven dried 100 mL round bottom double opening flask equipped with a magnetic stir bar taken Intermediate-30 (2.0 g, 2.9 mmol) and then suspended in 40 mL of Anhydrous toluene under argon atmosphere. G (1,2,4-Butanetriol, 0.94 g, 8.8 mmol) and PPTS (1.4 g, 5.9 mmol) were added and used a Dean-Stark device with heating and refluxing at 135 ℃for 20 h. The reaction was monitored by TLC. After the reaction was completed, it was cooled to room temperature. An equal volume of water was added to the reaction solution and then extracted with ethyl acetate (50 ml × 3). Solvents were removed in vacuo. The product was purified by SiO_2_ gel rapid column chromatography. Petroleum ether and ethyl acetate were used as eluents at 10:1 to obtain Intermediate-31, which was colorless oily liquid. Yield: 38%, 0.83 g.

KEL12: To an oven dried 25 mL round-bottom flask equipped with a magnetic stir bar taken Intermediate-31 (0.83 g, 1.11 mmol) and then suspended in 10 mL of an ultra-dry DCM under argon atmosphere at 0 ℃. Later, Methanesulfonic anhydride (0.39 g, 2.22 mmol) and Et_3_N (0.34 g, 3.33 mmol) were added and allowed to stir at room temperature for overnight. The reaction was monitored by TLC. After the reaction was completed, it was cooled to room temperature. Solvents were removed in vacuo. Crude Intermediate-32 were obtained. To an oven dried 50 mL round bottom double opening flask equipped with a magnetic stir bar taken crude Intermediate-32 under argon atmosphere. 20 ml of dimethylamine 2.0 M in THF solution and 0.5 ml Et_3_N were added and allowed to stir at room temperature for 72 h. The reaction was monitored by TLC. After the reaction was completed, an equal volume of water was added to the reaction solution and then extracted with ethyl acetate (50 ml × 3). Solvents were removed in vacuo. The product was purified by SiO_2_ gel rapid column chromatography. DCM and methyl alcohol add ammonia water were used as eluents at 40:1 to obtain KEL12, which was yellow oily liquid. Yield: 21%, 185 mg. ^1^H NMR (600 MHz, CDCl_3_) δ 4.82 – 4.80 (m, 1H), 4.03 (t, *J* = 6.6 Hz, 1H), 3.99 (t, *J* = 6.6 Hz, 3H), 3.42 (t, *J* = 7.6 Hz, 1H), 2.41 – 2.36 (m, 1H), 2.31 – 2.26 (m, 1H), 2.23 – 2.20 (m, 10H), 1.78 – 1.73 (m, 1H), 1.66 – 1.60 (m, 1H), 1.58 – 1.43 (m, 12H), 1.31 – 1.20 (m, 54H), 0.82 (t, *J* = 7.0 Hz, 9H); ^13^C NMR (151 MHz, CDCl_3_) δ 173.92, 173.60, 112.00, 74.75, 74.73, 74.05, 70.00, 64.31, 56.29, 45.41, 37.83, 37.73, 37.50, 37.42, 34.73, 34.40, 34.20, 31.93, 31.90, 31.73, 31.71, 29.82, 29.80, 29.58, 29.56, 29.53, 29.50, 29.33, 29.30, 29.26, 29.20, 28.67, 28.65, 25.96, 25.35, 25.19, 25.05, 23.90, 23.71, 23.59, 22.69, 14.13; MS-ESI (m/z): 794.7 (M+H)^+^.


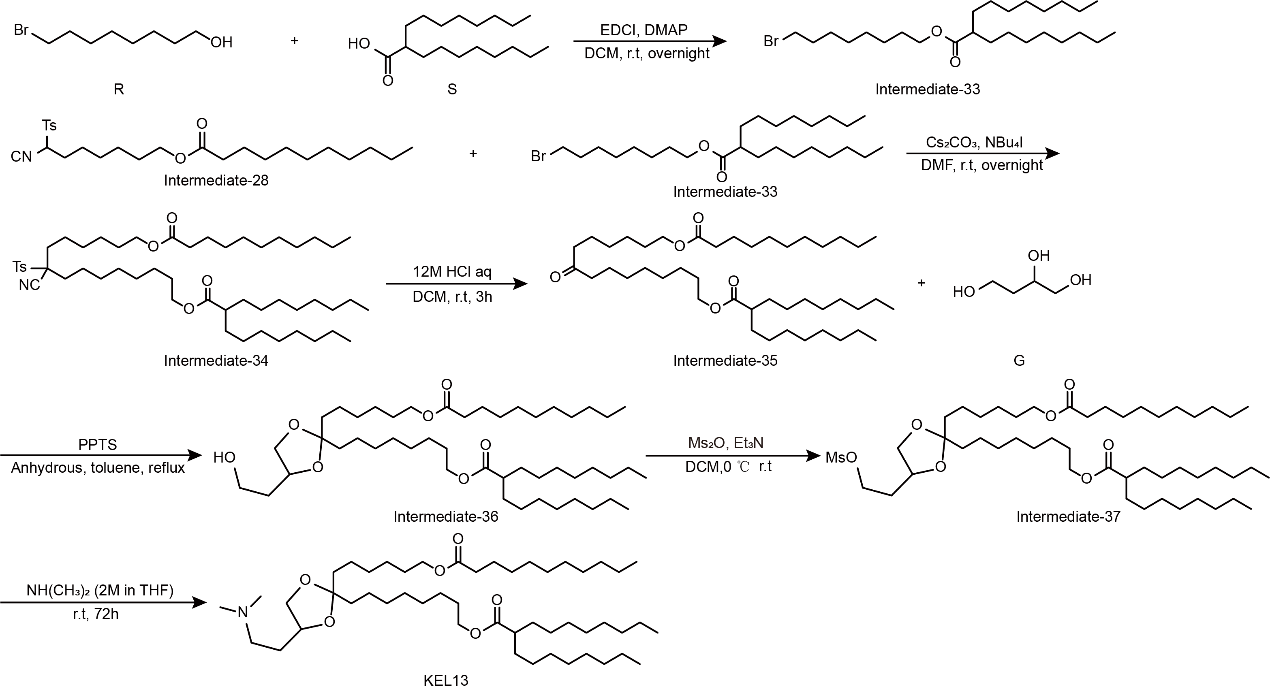


**Scheme S13.** Synthesis of KEL13.

**Synthesis of KEL13:** Intermediate-28: The synthesis of Intermediate-28 refers to the synthesis route of KEL12.

Intermediate-33: To an oven dried 500 mL round-bottom flask equipped with a magnetic stir bar taken S (2-Octyldecanoic acid, 10.0 g, 35.15 mol) and then suspended in 200 mL of DCM under argon atmosphere. EDCI (8.76 g, 45.70 mol) and DMAP (1.72 g, 14.06 mol) were then added and allowed to stir for 15 min. R (8-Bromo-1-octanol, 11.0 g, 52.73 mol) was then added and allowed to stir at room temperature for overnight. After the reaction was completed, an equal volume of saturated sodium bicarbonate solution was added to the reaction solution to dilute and separate, and anhydrous magnesium sulfate was added to the organic layer to dry, filter, and solvents were removed in vacuo. The product was purified by SiO_2_ gel rapid column chromatography. Petroleum ether and ethyl acetate were used as eluents at 30:1 to obtain intermediate-33, which was colorless oily liquid. Yield: 87%, 14.0 g.

Intermediate-34: To an oven dried 500 mL round-bottom flask equipped with a magnetic stir bar taken Intermediate-28 (27.0 g, 59.0 mmol) and then suspended in 200 mL of DMF under argon atmosphere. Intermediate-33 (14.0 g, 29.5 mmol), Cs_2_CO_3_ (28.1 g, 88.6 mmol) and NBu_4_I (5.5 g, 14.8 mmol) were then added and allowed to stir at room temperature for 12 h. The reaction was monitored by TLC. After the reaction was completed, an equal volume of water was added to the reaction solution, diluted, extracted with ethyl acetate (50 ml × 3), and solvents were removed in vacuo. The product was purified by SiO_2_ gel rapid column chromatography. Petroleum ether and ethyl acetate were used as eluents at 3:1 to obtain intermediate-34, which was colorless oily liquid. Yield: 76%, 19.0 g.

Intermediate-35: To an oven dried 250 mL round-bottom flask equipped with a magnetic stir bar taken Intermediate-34 (19.0 g, 22.2 mmol) and then suspended in 100 mL of DCM under argon atmosphere. 60 ml of 12 M HCl was added slowly and stirred for 3 h at room temperature. The reaction was monitored by TLC. After the reaction was completed, the liquid was separated, and the organic layer was washed with saturated sodium bicarbonate solution, dried with anhydrous magnesium sulfate, and solvents were removed in vacuo. The product was purified by SiO_2_ gel rapid column chromatography. Petroleum ether and ethyl acetate were used as eluents at 2:1 to obtain intermediate-35, which was colorless liquid. Yield: 91%, 14.0 g.

Intermediate-36: To an oven dried 100 mL round bottom double opening flask equipped with a magnetic stir bar taken Intermediate-35 (2.5 g, 3.6 mmol) and then suspended in 40 mL of Anhydrous toluene under argon atmosphere. G (1,2,4-Butanetriol, 0.76 g, 7.2 mmol) and PPTS (2.7 g, 10.8 mmol) were added and used a Dean-Stark device with heating and refluxing at 135 ℃for 20 h. The reaction was monitored by TLC. After the reaction was completed, it was cooled to room temperature. An equal volume of water was added to the reaction solution and then extracted with ethyl acetate (50 ml × 3). Solvents were removed in vacuo. The product was purified by SiO_2_ gel rapid column chromatography. Petroleum ether and ethyl acetate were used as eluents at 10:1 to obtain Intermediate-36, which was colorless oily liquid. Yield: 31%, 0.87 g.

KEL13: To an oven dried 25 mL round-bottom flask equipped with a magnetic stir bar taken Intermediate-36 (0.87 g, 1.11 mmol) and then suspended in 10 mL of an ultra-dry DCM under argon atmosphere at 0 ℃. Later, Methanesulfonic anhydride (0.39 g, 2.22 mmol) and Et_3_N (0.34 g, 3.33 mmol) were added and allowed to stir at room temperature for overnight. The reaction was monitored by TLC. After the reaction was completed, it was cooled to room temperature. Solvents were removed in vacuo. Crude Intermediate-37 was obtained. To an oven dried 50 mL round bottom double opening flask equipped with a magnetic stir bar taken crude Intermediate-37 under argon atmosphere. 20 ml of dimethylamine 2.0 M in THF solution and 0.5 ml Et_3_N were added and allowed to stir at room temperature for 72 h. The reaction was monitored by TLC. After the reaction was completed, an equal volume of water was added to the reaction solution and then extracted with ethyl acetate (50 ml × 3). Solvents were removed in vacuo. The product was purified by SiO_2_ gel rapid column chromatography. DCM and methyl alcohol add ammonia water were used as eluents at 40:1 to obtain KEL13, which was yellow oily liquid. Yield: 55%, 498 mg. ^1^H NMR (500 MHz, CDCl_3_) δ 4.07 – 4.00 (m, 6H), 3.44 (t, *J* = 7.5 Hz, 1H), 2.42 – 2.36 (m 1H), 2.32 – 2.21 (m, 10H), 1.81 – 1.74 (m, 1H), 1.68 – 1.51 (m, 14H), 1.42 – 1.34 (m, 2H), 1.26 – 1.22 (m, 53H), 0.84 (t, *J* = 6.9 Hz, 9H); ^13^C NMR (126 MHz, CDCl_3_) δ 176.69, 173.97, 112.05, 74.81, 70.06, 64.36, 64.16, 56.36, 45.92, 45.50, 37.93, 37.78, 37.59, 37.48, 34.46, 32.63, 31.97, 31.94, 31.82, 29.63, 29.53, 29.37, 29.32, 29.24, 28.81, 28.71, 27.54, 26.06, 26.02, 25.09, 24.07, 23.94, 23.77, 23.64, 22.73, 14.16; MS-ESI (m/z): 808.7 (M+H)^+^.

**2.2. Purity determination and chiral separation**

**Purity determination of KEL lipids:** Target compounds were performed on a Thermo Vanquish HPLC-CAD system.Conditions (Mobile phase: solvent A = H_2_O + 0.3% triethylamine+0.15% acetic acid; solvent B = Methanol + 0.3% triethylamine+0.15% acetic acid ); Waters XSelect CSH C18 column (150 mm × 4.6 mm, 3.5 μm, PN: 186005270 ); injection volumn: 20 µL; column temperature : 55℃; flow: 0.8 mL/min; CAD Date rate: 10 Hz; Filter: 3.6 s, evaporative temperature: 35℃, the gradient table is as below:

| Time(min) | solvent A (%) | solvent B (%) |
| --- | --- | --- |
| 0 | 4 | 96 |
| 45 | 0 | 100 |
| 60 | 0 | 100 |
| 60.1 | 4 | 96 |
| 65 | 4 | 96 |

**Chiral purity determination:** Target compounds were performed on a Thermo Vanquish HPLC-CAD system. Conditions (Mobile phase: solvent A = H_2_O + 0.3% triethylamine+0.15% acetic acid; solvent B = Methanol + 0.3% triethylamine+0.15% acetic acid ); DAICEL CHIRALPAK IA column (250 mm × 4.6 mm, 5 μm, PN: 80325 ); injection volumn: 20 µL; column temperature: 40℃; flow: 0.6 mL/min; CAD Date rate: 10 Hz; Filter: 3.6 s, evaporative temperature: 35℃, the gradient table is as below:

| Time(min) | solvent A (%) | solvent B (%) |
| --- | --- | --- |
| 0 | 4 | 96 |
| 30 | 0 | 100 |
| 40 | 0 | 100 |
| 40.1 | 4 | 96 |
| 50 | 4 | 96 |

**HPLC chiral separation of (*4S*)-KEL12:** Chiral separation was performed on a K-Prep LAB Preparative LC systems. Conditions: Methanol : Diethylamine = 100 : 0.1; CHIRALPAK® IA (5 cm × 25 cm, 10 μm); flow: 60 mL/min; UV at 214 nm; Temperature: 38 ^o^C.
